# Mapping Bacteriophage-Based Antimicrobial Research in Southeast Asia: A Bibliometric Analysis of Scientific Productivity, Knowledge Structure, and Emerging Research Pathways

**DOI:** 10.3390/biology15181596

**Published:** 2026-09-10

**Authors:** Christian Joseph N. Ong, Johnalie N. Cabalbag, Jowi Tsidkenu Pili Cruz, Teodora T. Valderama, Celia R. Roslin, Carina M. Magtibay, Janice Kaylyn K. Lonogan, Amelda C. Libres, Joel G. Matamis, Jose Edwardo R. Mamaat, Ferdinand A. Mortel, Grace D. Bacalzo, Kevin Smith P. Cabuhat, Carlos S. de Leon, Jose Jurel M. Nuevo, Joel R. Aguilan, Jamil Allen G. Fortaleza

**Affiliations:** 1 Department of Biology, College of Science, De La Salle University, Manila 1004, Philippines; christian_joseph_ong@dlsu.edu.ph (C.J.N.O.); johnalie_cabalbag@dlsu.edu.ph (J.N.C.); jowi.cruz@dlsu.edu.ph (J.T.P.C.); 2 Department of Biology, College of Arts and Sciences, Manila Central University, Caloocan 1400, Philippines; 3 Department of Medical Technology, College of Nursing, Cavite State University, Indang 4122, Philippines; teodora.valderama@cvsu.edu.ph; 4 School of Medical Technology, Centro Escolar University, Manila 1001, Philippines; crroslin@ceu.edu.ph; 5 College of Allied Medical Professions, Lyceum of the Philippines University, Batangas 4200, Philippines; cmmagtibay@lpubatangas.edu.ph; 6 School of Natural Sciences, University of Baguio, Baguio City 2600, Philippines; janub95@e.ubaguio.edu; 7 College of Medical Laboratory Science, Liceo de Cagayan University, Cagayan de Oro City 9000, Philippines; alibres@liceo.edu.ph; 8 School of Medical Laboratory Sciences, St. Dominic College of Asia, Bacoor 4102, Philippines; jgmatamis@sdca.edu.ph; 9 Department of Medical Technology, Far Eastern University, Manila 1008, Philippines; jemamaat@feu.edu.ph; 10 College of Medical Technology, Manila Central University, Caloocan 1400, Philippines; ferdinandmortel@gmail.com; 11 College of Allied Medical Sciences, Wesleyan University-Philippines, Cabanatuan City 3100, Philippines; gracebacalzo123@gmail.com; 12 Basic Education Department, La Consolacion University Philippines, Malolos 3000, Philippines; kevin_smith_cabuhat@dlsu.edu.ph; 13 Bulacan State University, Malolos City 3000, Philippines; 14 Department of Medical Laboratory Science, Dr. Carlos S. Lanting College, Quezon City 1116, Philippines; carlosdeleon@lanting.ph.education; 15 College of Medical Laboratory Science, Our Lady of Fatima University, Valenzuela City 1440, Philippines; jmnuevo@fatima.edu.ph; 16 College of Medical Technology, Calayan Educational Foundation, Inc., Lucena City 4301, Philippines; joel.aguilan@cefi.edu.ph; 17 National University, Sampaloc, Manila 1008, Philippines; 18 School of Arts and Sciences, NU Fairview, Quezon City 1100, Philippines

**Keywords:** bacteriophage, research trends, phage therapy, antimicrobial resistance, Southeast Asia, bibliometric analysis

## Abstract

Southeast Asia faces a substantial burden of antimicrobial resistance (AMR), creating increasing interest in bacteriophage-based antimicrobial strategies. However, the regional development, contributors, collaboration patterns, and evolving research priorities of this field remain insufficiently characterized. This study conducted a bibliometric analysis of Scopus-indexed publications on bacteriophage-based antimicrobial research affiliated with Southeast Asian countries from 1981 to 2025. Bibliometrix/Biblioshiny and VOSviewer were used to evaluate scientific production, citation patterns, leading contributors, collaboration networks, and conceptual and thematic development. A total of 862 publications were analyzed, with scientific output accelerating markedly after 2019 and reaching its highest level in 2025. Thailand emerged as the dominant regional contributor, with Vongkamjan (n = 21) and Surachat (n = 20) as the two most prolific authors. Mahidol University (n = 182), Prince of Songkla University (n = 142), and Universiti Putra Malaysia (n = 139) were the leading institutions, demonstrating a concentration of research capacity in Thailand and Malaysia. Scientific Reports was the most productive journal (30 publications), whereas Frontiers in Microbiology recorded the highest citation count among the leading sources (963 citations). Collaboration mapping revealed increasingly interconnected regional and international research networks. Thematic analyses demonstrated a transition from foundational and pathogen-specific investigations toward AMR, bacteriophage therapy, biofilm control, aquaculture, genomic and comparative genomic analysis, wastewater and public-health applications, and One Health-oriented research. Emerging topics included endolysins, quorum sensing, CRISPR-associated approaches, metagenomics, and genome-informed phage characterization. These findings demonstrate the rapid expansion and thematic diversification of bacteriophage-based antimicrobial research in Southeast Asia while highlighting persistent geographic concentration and the need for stronger regional infrastructure, collaboration, and translational capacity.

## Simple Summary

Bacteriophage research has attracted increasing attention in Southeast Asia as a potential strategy to address antimicrobial resistance and other bacterial threats. This study examines the development of research in the region and identifies emerging interests in phage therapy, biofilm control, aquaculture, genomics, and One Health. The results underscore the necessity for enhanced regional collaboration, shared research infrastructure, and increased efforts to translate phage research into practical and clinical applications.

## 1. Introduction

Antimicrobial resistance (AMR) is recognized as one of the most pressing public health threats in Southeast Asia (SEA). The rapid spread of multidrug-resistant (MDR) pathogens increasingly undermines therapeutic efficacy and healthcare sustainability. The region faces escalating burdens of resistant bacterial strains across clinical, agricultural, and environmental domains [1]. Factors such as antibiotic misuse, unrestricted access to antimicrobials, insufficient stewardship, and inadequate infection-control systems have accelerated the emergence and persistence of resistant pathogens [2,3]. Environmental contamination, hospital wastewater, intensive livestock production, and densely populated urban areas also facilitate the regional transmission of AMR through interconnected One Health pathways [4,5]. These combined healthcare and ecological factors have established SEA as a global hotspot for antimicrobial resistance. This situation highlights the urgent need for alternative antimicrobial interventions [6].

Considering the diminishing effectiveness of conventional antibiotics, bacteriophages have re-emerged as promising therapeutic alternatives for MDR bacterial infections. Bacteriophages are viruses that specifically infect bacterial hosts. They adsorb to the host cell surface, replicate intracellularly, and cause bacterial lysis. Although phage therapy predates the antibiotic era and was historically employed in Eastern Europe and the former Soviet Union, its global development declined after the widespread adoption of broad-spectrum antibiotics [7]. The escalating AMR crisis has renewed scientific and clinical interest in phage therapeutics. Phages offer several advantages, including host specificity, self-replicating antibacterial activity, biofilm disruption, and minimal impact on the normal microbiota [8]. Additionally, phages can be engineered or combined into phage cocktails to expand their host range and reduce the development of resistance. Phage-derived enzymes such as endolysins further enhance their translational potential [9]. Advances in genome sequencing, synthetic biology, and molecular characterization have also improved the feasibility of phage-based interventions in contemporary antimicrobial therapy [10].

SEA is a strategically significant region for bacteriophage research due to its exceptional biodiversity, tropical ecosystems, extensive aquatic environments, and high burden of infectious diseases. Various ecological reservoirs, such as rivers, coastal waters, sewage systems, aquaculture facilities, agricultural settings, and hospital effluents, provide abundant sources for the discovery and characterization of novel phages. The region also faces significant challenges posed by foodborne infections, healthcare-associated pathogens, and antibiotic-resistant bacteria. These challenges create strong incentives for alternative antimicrobial strategies [11]. In recent years, scientific output related to bacteriophages and phage therapy has increased across ASEAN countries. Research has particularly grown in food safety, aquacultue, bacterial genomics, and antimicrobial applications. However, research capacity remains unevenly distributed. Disparities in sequencing infrastructure, bioinformatics expertise, laboratory standardization, funding, and translational readiness limit broader regional progress in phage therapeutics [12]. Substantial knowledge gaps remain regarding the overall intellectual structure and translational trajectory of bacteriophage research in SEA. Existing investigations are frequently fragmented across microbiology, aquaculture, environmental science, food safety, and clinical medicine. This fragmentation results in limited integration of regional evidence. Much of the current research remains concentrated on phage isolation, host-range characterization, and in vitro antibacterial activity. Comparatively fewer studies progress toward in vivo validation, clinical application, pharmacokinetic evaluation, or manufacturing standardization [13]. Regulatory uncertainty, insufficient biobanking infrastructure, lack of harmonized quality-control frameworks, and limited regional collaboration further impede the clinical translation of phage therapy. Similar limitations have also been observed in other low- and middle-income settings, where inadequate infrastructure and regulatory preparedness hinder the development of phage-based therapeutics despite increasing scientific interest [14,15].

Therefore, this study aimed to map the scientific development of bacteriophage-based antimicrobial research in SEA through bibliometric analysis. Specifically, it examined publication and citation trends, leading authors and institutions, collaboration networks, thematic evolution, and emerging research directions within the regional literature on phage-based antibacterial applications. Particular attention was given to research involving antimicrobial resistance, phage therapy, biofilm control, clinically and economically important bacterial pathogens, aquaculture and food-safety applications, genomic characterization, and emerging One Health perspectives. By defining the scope according to the antimicrobial and therapeutic orientation of the search strategy, this study provides a focused assessment of how Southeast Asian phage research has developed in response to bacterial disease and antimicrobial-resistance challenges. The analysis further identifies translational, infrastructural, and regulatory priorities that may influence the future development of phage-based antimicrobial innovation across the region.

## 2. Materials and Methods

### 2.1. Study Design

This study employed a bibliometric research design combining performance analysis and science mapping to characterize the development of bacteriophage-based antimicrobial research in SEA. Bibliographic metadata were quantitatively analyzed to examine publication and citation patterns, productive authors and institutions, scientific collaboration, keyword relationships, and thematic evolution. Unlike a systematic review, which is designed to comprehensively identify and critically synthesize evidence addressing a predefined research question, the present study aimed to characterize the structure, productivity, collaboration patterns, and conceptual development of a defined scholarly literature. Individual publications were therefore not assessed for methodological quality, risk of bias, or clinical evidence strength. Record screening and filtering were performed to ensure that the bibliographic dataset corresponded to the predefined research domain and to minimize irrelevant database retrievals before performance analysis and science mapping.

### 2.2. Data Source

Scopus was selected as the sole bibliographic source for its extensive indexing of journals in phage biology and applied microbiology, robust citation-tracking capabilities, and widespread use in scientometric research. Its comparatively broad coverage of applied microbiology and bacteriophage-related publications makes it particularly appropriate for examining the development of phage research in SEA. Restricting data retrieval to a single database also promotes metadata consistency and minimizes duplicate records. Although combining multiple databases may marginally expand coverage, it often introduces discrepancies in citation counts, author names, institutional affiliations, and keyword indexing. Resolving these inconsistencies requires substantial data harmonization and may introduce additional bias due to incorrect record matching, fragmented author profiles, or divided citation totals. The use of a single comprehensive source, therefore, supports more standardized bibliometric fields, strengthens the reliability of network analyses, and improves methodological reproducibility.

This database selection is consistent with established scientometric practice, in which the primary objective is to map the intellectual, collaborative, and thematic structure of a field rather than to achieve the exhaustive literature coverage expected in a systematic review. Scopus also enables the export of detailed bibliographic information, including publication year, authorship, institutional affiliation, journal source, author and indexed keywords, abstract, subject classification, citation count, and source-level indicators such as CiteScore. These data provide the basis for assessing publication performance, collaboration patterns, influential sources, and thematic development.

### 2.3. Search Strategy and Data Extraction

**Figure 1:**
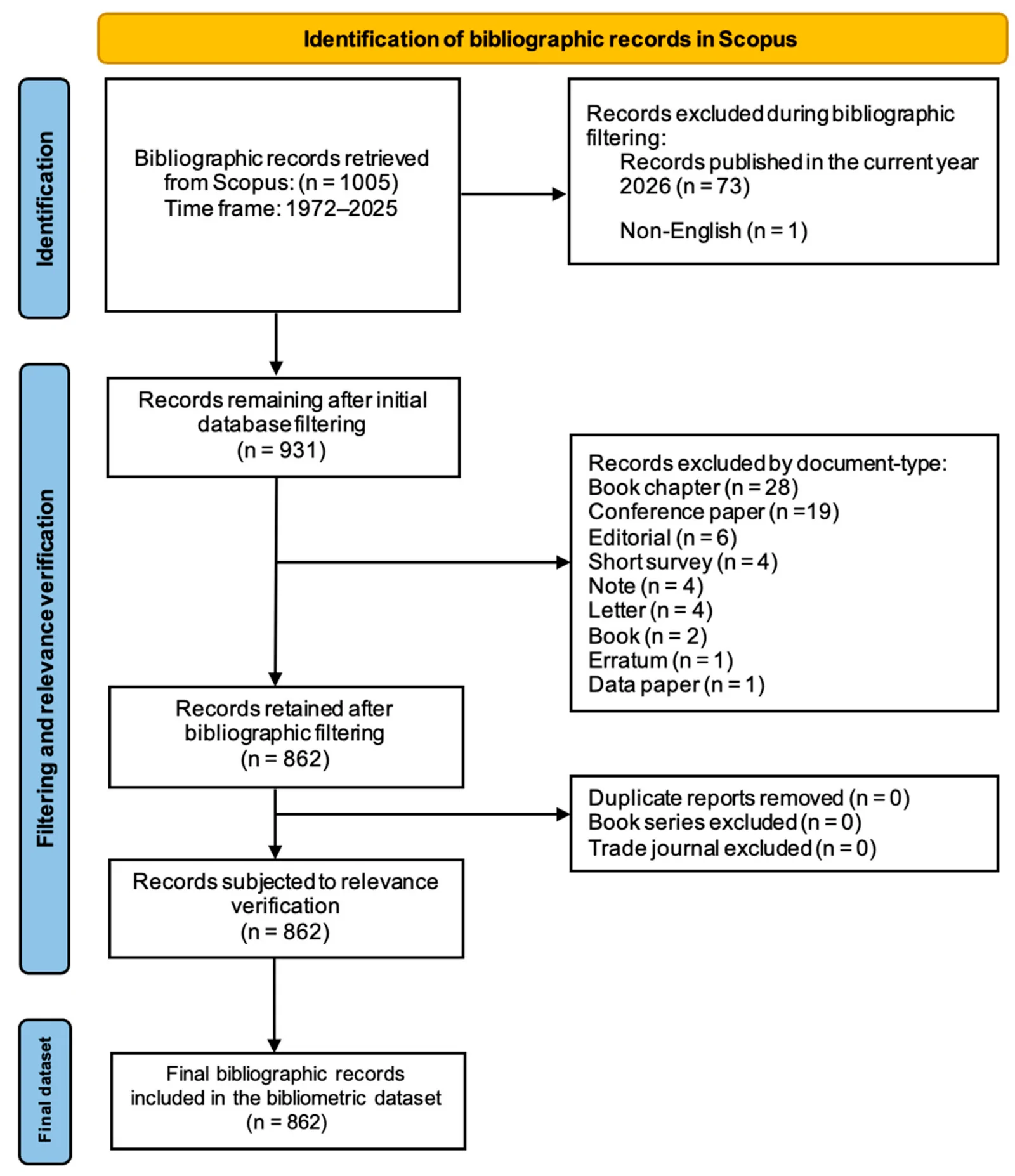
Bibliographic record retrieval, filtering, and dataset construction for the bibliometric analysis of bacteriophage-based antimicrobial research in SEA. Records retrieved from Scopus were subjected to predefined publication-year, language, document-type, and relevance criteria before construction of the final bibliographic dataset. The flow structure was adapted from PRISMA solely to enhance transparency in reporting database retrieval and filtering and should not be interpreted as a systematic-review study-selection process.

Bibliographic records were retrieved from Scopus on 24 May 2026 using the TITLE-ABS-KEY field. Following database retrieval, records were subjected to predefined bibliographic filtering and relevance screening to construct the final dataset used for performance analysis and science mapping. A PRISMA-inspired flow diagram was used solely as a transparent visual framework for documenting the number of records retrieved, excluded during bibliographic filtering and relevance screening, and retained for bibliometric analysis (Figure 1). Its use does not imply that the present investigation constitutes a PRISMA-compliant systematic review or that included publications underwent systematic evidence synthesis, risk-of-bias assessment, or evaluation of intervention outcomes. Rather, the diagram was adapted to improve transparency and reproducibility in reporting the construction of the bibliometric dataset.

The query therefore combined broad bacteriophage terminology (“bacteriophage*,” “bacteriophag*,” and “phage*”) with application-specific terms such as “bacteriophage therapy,” “phage therapy,” “phage-based therapy,” “therapeutic phage*,” “phage cocktail*,” “lytic phage*,” and “bacteriophage treatment.” These terms were required to co-occur with antimicrobial-context descriptors, including “antimicrobial*,” “antibacterial*,” “bacterial infection*,” “drug-resistant bacteria,” “AMR,” “MDR bacteria,” “superbug*,” or “biofilm*.” This combination was used to increase specificity for literature in which bacteriophages were investigated in relation to bacterial control or antimicrobial applications while reducing retrieval of phage studies unrelated to the study objectives. Consequently, the resulting dataset should be interpreted as representing the bacteriophage-based antimicrobial research landscape in SEA rather than the complete regional literature on bacteriophage biology. Within this defined scope, the strategy captures interconnected areas including phage therapy, antibacterial and antibiofilm applications, AMR-related investigations, pathogen biocontrol, aquaculture and food-safety applications, and genomic or molecular characterization conducted in an antimicrobial context.

To improve data stability and citation comparability, publications indexed in 2026 were excluded because the year was incomplete at the time of data retrieval and records had insufficient time for citation accrual. The dataset was restricted to peer-reviewed original research articles and review articles published in English-language journals to maintain consistency in bibliometric indicators and document characteristics. Editorials, letters, conference proceedings, notes, book chapters, and other document types were excluded. These criteria were applied to construct a standardized bibliographic dataset suitable for performance analysis and science mapping rather than to determine evidence eligibility for a systematic review. Accordingly, the final corpus represents Scopus-indexed literature within the predefined domain of bacteriophage-based antimicrobial research in SEA and should not be interpreted as an exhaustive representation of all fundamental or applied bacteriophage research conducted in the region. Eligible bibliographic records were exported in CSV format with complete metadata for subsequent data cleaning and bibliometric analysis. Journal-level CiteScore metrics were obtained from Scopus to contextualize source-level influence.

The search strategy was intentionally designed to delineate a specific bibliometric domain, bacteriophage-based antimicrobial research, rather than to retrieve the complete literature on bacteriophage biology in SEA. Broad phage-related terms (“bacteriophage*,” “bacteriophag*,” and “phage*”) were therefore combined with antimicrobial- and bacterial-control descriptors to increase specificity for studies in which bacteriophages were investigated in relation to antimicrobial activity, bacterial infection, antimicrobial resistance, biofilms, or pathogen control. This design is consistent with the stated objective and title of the study, which focus specifically on the antimicrobial research landscape. An important consequence of this operational definition is that fundamental studies of phage ecology, taxonomy, evolution, phage-host interactions, environmental virology, or phage genomics may not have been retrieved when they lacked an explicit antimicrobial or bacterial-control context. Accordingly, the thematic distributions reported in this study should not be interpreted as representing the complete bacteriophage research landscape of SEA. Rather, they characterize the intellectual structure and thematic evolution of the antimicrobial-oriented subset defined a priori by the search strategy. Genomic, ecological, environmental, or phage-host interaction studies appearing in the present analysis should therefore be interpreted as those connected to this antimicrobial context rather than as comprehensive representations of their respective fundamental research domains.

The Scopus advanced search query was designed to identify scholarly articles on research on phage and was applied to article titles and abstracts. The complete search string utilized in this study is provided below:

TITLE-ABS-KEY (“bacteriophage therapy” OR “phage therapy” OR bacteriophage* OR bacteriophag* OR phage* OR “phage-based therapy” OR “therapeutic phage*” OR “phage cocktail*” OR “lytic phage*” OR “bacteriophage treatment”) AND (antimicrobial* OR antibacterial* OR “bacterial infection*” OR “drug-resistant bacteria” OR AMR OR “MDR bacteria” OR superbug* OR biofilm*) AND AFFILCOUNTRY (“Philippines” OR “Thailand” OR “Malaysia” OR “Myanmar” OR “Timor Leste” OR “Timor-Leste” OR “East Timor” OR “Cambodia” OR “Singapore” OR “Indonesia” OR “Laos” OR “Viet Nam” OR “Brunei Darussalam”) AND (LIMIT-TO (DOCTYPE, “ar”) OR LIMIT-TO (DOCTYPE, “re)) AND (EXCLUDE (PUBYEAR, “2026”)) AND (LIMIT-TO (LANGUAGE, “English”))

### 2.4. Data Cleaning and Harmonization

Manual data cleaning was conducted to improve the accuracy, consistency, and interpretability of the bibliometric dataset prior to analysis. The procedure included removal of duplicate records, standardization of author and institutional names, and harmonization of author keywords using a manually curated thesaurus file. Synonymous or closely equivalent terms referring to the same concept were consolidated to prevent fragmentation of conceptually identical nodes, including “bacteriophage,” “bacteriophages,” “phage,” “lytic bacteriophage,” and “phages”; “bacteriophage therapy” and “phage therapy”; “antimicrobial resistance,” “multidrug resistance,” and “antibiotic resistance”; “antimicrobials,” “antimicrobial,” “antibiotics,” and “antibiotic”; “biofilm” and “biofilms”; “*Escherichia coli*” and “*E. coli*”; and “microbiome” and “microbiota.” Minimum thresholds for network analyses were set at five keyword occurrences and ten institutional publications to reduce low-frequency analytical noise while retaining recurrent patterns within the dataset.

For thematic keyword analyses, selected terms that did not represent substantive research concepts were excluded using a predefined relevance criterion. Geographic descriptors such as “Vietnam” and “Thailand” were removed because they primarily indicated the location or affiliation context of publications rather than a scientific topic, method, organism, intervention, or research problem. Their retention in keyword co-occurrence and conceptual-structure analyses could cause geographic frequency to influence cluster formation without providing additional information about the intellectual content of the literature. Importantly, these geographic terms were removed only from thematic keyword-based analyses and were retained in the underlying bibliographic dataset and in all country productivity and collaboration analyses. Their exclusion therefore did not alter country-level publication counts or co-authorship relationships.

Highly generic descriptors such as “bacteria,” “bacterial,” and “pathogen(s)”, including “biocontrol” were excluded from conceptual mapping because these terms broadly characterize the biological targets of bacteriophages but provide limited discriminatory information regarding specific research themes, applications, mechanisms, or host systems. Their high generality could disproportionately influence keyword frequency and network structure without identifying a distinct intellectual domain. Importantly, this exclusion did not remove publications involving bacterial pathogens from the bibliographic corpus. Instead, more informative organism- and application-specific terms, including *Escherichia coli*, *Salmonella*, *Staphylococcus aureus*, *Pseudomonas aeruginosa*, *Klebsiella pneumoniae*, *Acinetobacter baumannii*, “antimicrobial resistance,” “biofilm,” “aquaculture,” and related concepts, were retained when they satisfied the predefined analytical threshold. Other highly generic or non-conceptual terms were removed only when they lacked sufficient specificity to differentiate research themes. The cleaning procedure was applied consistently on the basis of conceptual relevance rather than country productivity or the direction of the resulting thematic clusters, thereby reducing the potential for selective interpretation.

### 2.5. Data Analysis and Tools

The retrieved bibliographic dataset (.csv) was analyzed using a combination of Bibliometrix/Biblioshiny version 5.0 (R package) [15] and VOSviewer version 1.6.20 (Universiteit Leiden and CWTS Meaningful Metrics) to perform complementary performance analysis and science mapping. Bibliometrix/Biblioshiny was employed to generate descriptive bibliometric indicators, including annual scientific production, citation trends, leading journals, prolific authors, productive institutions, and thematic trend analyses. In parallel, VOSviewer was used to construct and visualize bibliometric networks, including co-authorship, institutional and international collaboration, and keyword co-occurrence, thereby revealing the structural organization and intellectual relationships within the research domain. Together, these platforms provided complementary analytical capabilities for evaluating both research performance and the conceptual architecture of the field.

The network analyses were primarily designed for bibliometric science mapping and visualization of co-authorship, institutional collaboration, international collaboration, and keyword co-occurrence patterns rather than for formal social-network analysis. Accordingly, interpretations were based on node size, link structure, cluster membership, and the relative configuration of nodes within the VOSviewer-generated networks. Quantitative node-level measures such as total link strength and graph-theoretic centrality indices were not included because these parameters could not be consistently retrieved or reconstructed from the finalized analytical outputs. To avoid introducing potentially non-reproducible estimates, the study therefore retained a descriptive network-mapping approach and interpreted apparent network prominence cautiously rather than treating visual position as a formal measure of centrality.

For author- and institution-level productivity analyses, a full-counting approach was applied, whereby each publication was credited to every contributing author and each participating institutional affiliation represented in the bibliographic record. This approach was selected because the objective of these analyses was to characterize research participation and identify the most productive contributors within the regional literature rather than to estimate fractional contributions to individual publications. Accordingly, author and institutional rankings were based on total publication output and should be interpreted as indicators of scientific productivity rather than individual or institutional research impact.

Citation-based indicators were evaluated separately through temporal citation analysis and source-level metrics, including total citations, average citations per publication, and h-index. Author- and institution-specific citation indicators were not incorporated into the productivity rankings because citation accumulation is strongly influenced by publication age, disciplinary citation practices, collaboration patterns, and a small number of highly cited publications. Maintaining publication productivity and citation impact as analytically distinct dimensions also avoids conflating research participation with citation influence. Thus, the present analysis uses publication counts to characterize the distribution of author and institutional productivity, while citation analyses provide complementary evidence of the broader scholarly visibility and influence of the analyzed literature.

**Figure 2:**
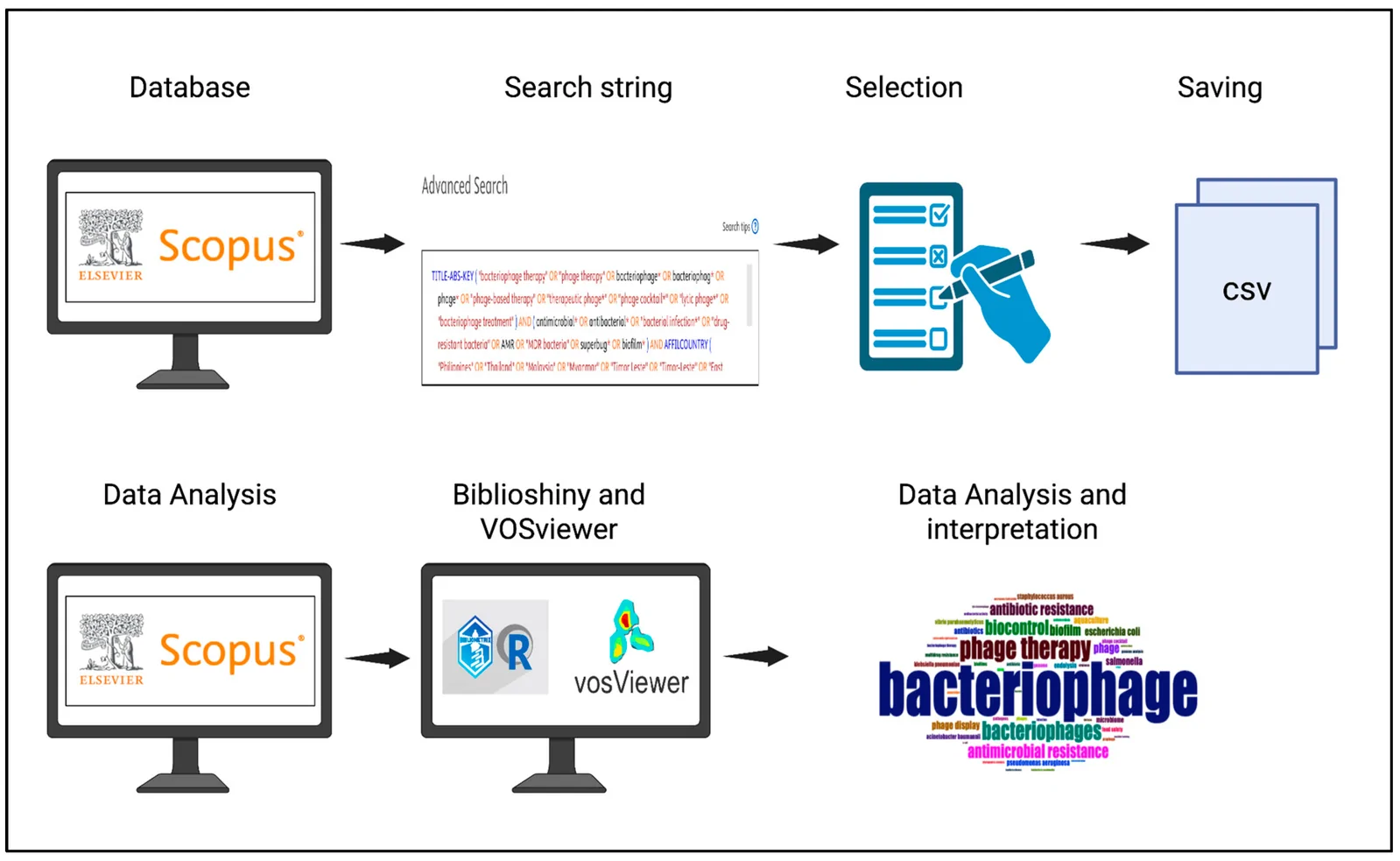
Schematic workflow of the bibliometric analysis conducted in this study, illustrating the processes of database retrieval from Scopus, search strategy formulation, study selection and data exportation, followed by enrichment and science mapping analyses using Bibliometrix/Biblioshiny (R package) and VOSviewer for visualization.

To investigate the evolution of research themes, keyword co-occurrence analyses derived from titles, abstracts, and author keywords were conducted to generate network clusters, temporal overlay visualizations, strategic thematic maps, thematic evolution diagrams, and multidimensional scaling (MDS) plots. These visualizations enabled the identification of core research topics, emerging themes, and shifts in scientific focus over time. The overall scientometric workflow is summarized in Figure 2, illustrating the sequential process from database retrieval and literature selection to metadata export, data preprocessing, bibliometric analysis using Bibliometrix/Biblioshiny and VOSviewer, and the subsequent interpretation of research performance, collaboration patterns, and thematic development.

## 3. Results

### 3.1. Annual Publication, Citation Trends, and Temporal Dynamics

**Figure 3:**
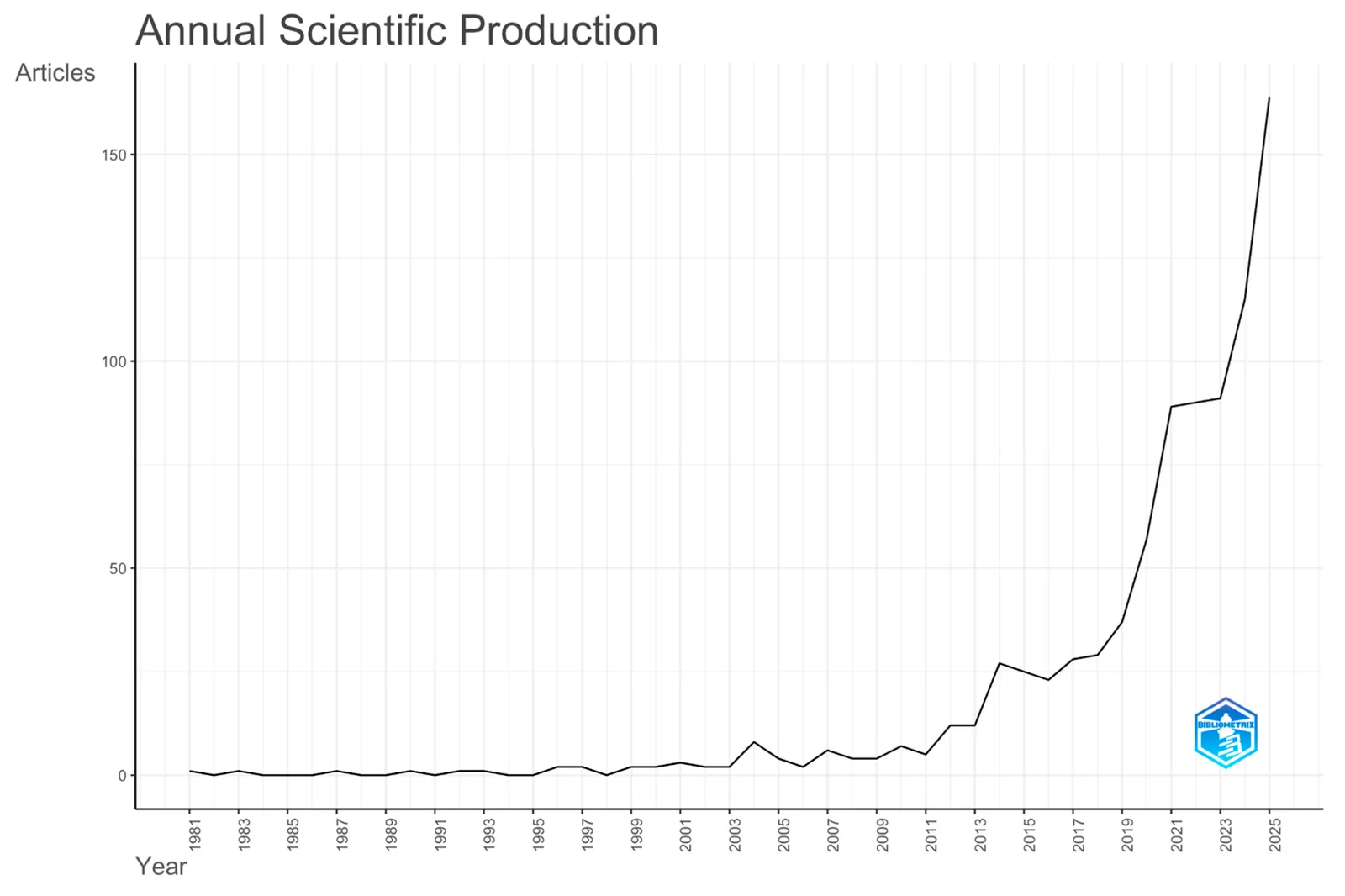
Trend analysis of Southeast Asian scientific publications on bacteriophage-based antimicrobial research in SEA.

A total of 862 documents, including articles and reviews, were retrieved and analyzed from the Scopus database. Annual scientific output demonstrates that bacteriophage research in SEA was sparse and episodic from 1981 to the early 2000s, with publication numbers remaining consistently low (Figure 3). This extended period of limited activity suggests that phage-related studies were initially peripheral to the region, primarily confined to isolated microbiological, environmental, or diagnostic investigations rather than constituting a cohesive research field. A modest increase in publications emerged after the mid-2000s, followed by a more pronounced upward trend after 2012–2014. This pattern indicates a gradual consolidation of regional interest in bacteriophages, particularly as antimicrobial resistance, food safety, aquaculture health, and pathogen surveillance became increasingly important research priorities.

A marked acceleration in annual publications is evident from 2019 onward, culminating in the highest output in 2025. This pronounced growth indicates that bacteriophage research in SEA has evolved from a niche subject to a rapidly expanding field, likely driven by renewed global interest in phage therapy, antibiotic alternatives, One Health surveillance, and biofilm-associated infections. The particularly strong increase after 2020 may be attributed to enhanced sequencing capacity, increased regional engagement in AMR-focused research, and the growth of collaborative networks. Collectively, these trends illustrate a rapidly maturing scientific landscape in which SEA is playing an increasingly significant role in bacteriophage research relevant to clinical microbiology, agriculture, aquaculture, and environmental health.

**Figure 4:**
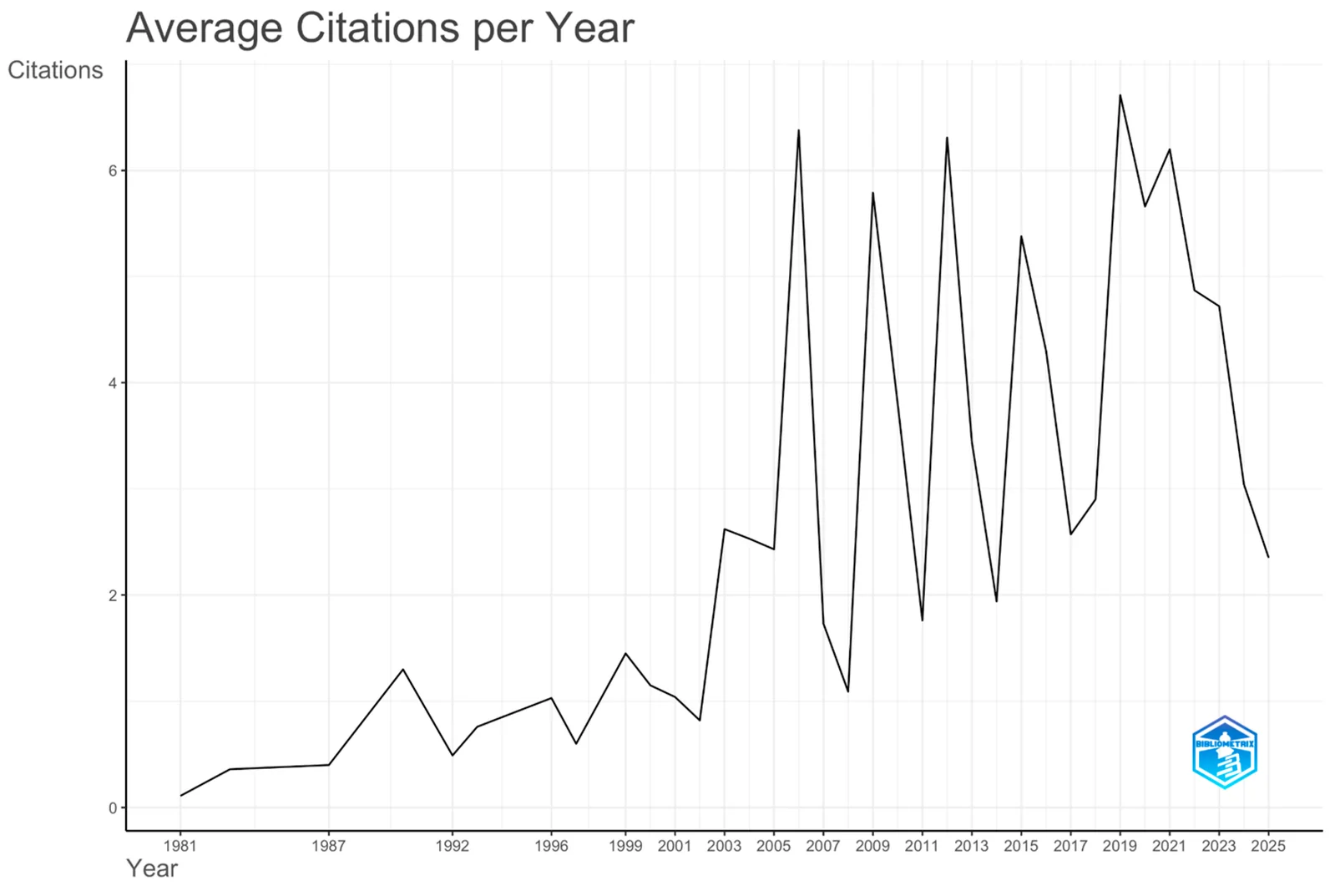
Citation trend analysis of bacteriophage-based antimicrobial research in SEA.

Citation trends indicate that bacteriophage-based antimicrobial research in SEA exhibited relatively limited citation activity during the earlier decades of the study period, with average annual citations generally remaining below two per article from 1981 through the early 2000s (Figure 4). A more pronounced increase became evident after 2003, accompanied by greater year-to-year variation in citation activity. However, these temporal differences should be interpreted cautiously because citation accumulation is inherently influenced by publication age, annual publication volume, disciplinary citation practices, and the presence of individual highly cited papers. Consequently, higher citation values among earlier publication cohorts may partly reflect their longer exposure to the scholarly literature rather than intrinsically greater scientific influence. Conversely, lower citation values among more recent publications should not be interpreted as evidence of reduced relevance or impact because these studies have had substantially less time to accumulate citations.

From 2006 onward, the citation profile became increasingly variable, with recurring peaks exceeding five to six average citations per year in several publication cohorts, including 2006, 2009, 2012, 2015, and 2019–2021. These fluctuations indicate heterogeneity in citation performance and may reflect the influence of particularly visible publications within years characterized by different levels of scientific output. Although the relatively strong citation activity observed for some recent cohorts coincides with the expansion of regional research on antimicrobial resistance, phage therapy, aquaculture, food safety, and environmental microbiology, this pattern should not be interpreted as a direct measure of increasing scientific or translational impact without accounting for differences in citation exposure time. In particular, the apparent decline in citation indicators for publications from 2024 to 2025 is expected given their substantially shorter citation windows at the time of data extraction. Accordingly, the citation trajectory is best interpreted as evidence of changing scholarly visibility and citation activity within the field rather than as a direct chronological ranking of research impact. Raw citation counts and average citation measures should therefore be considered alongside publication age and other bibliometric indicators when evaluating the development and influence of bacteriophage-based antimicrobial research in SEA.

**Figure 5:**
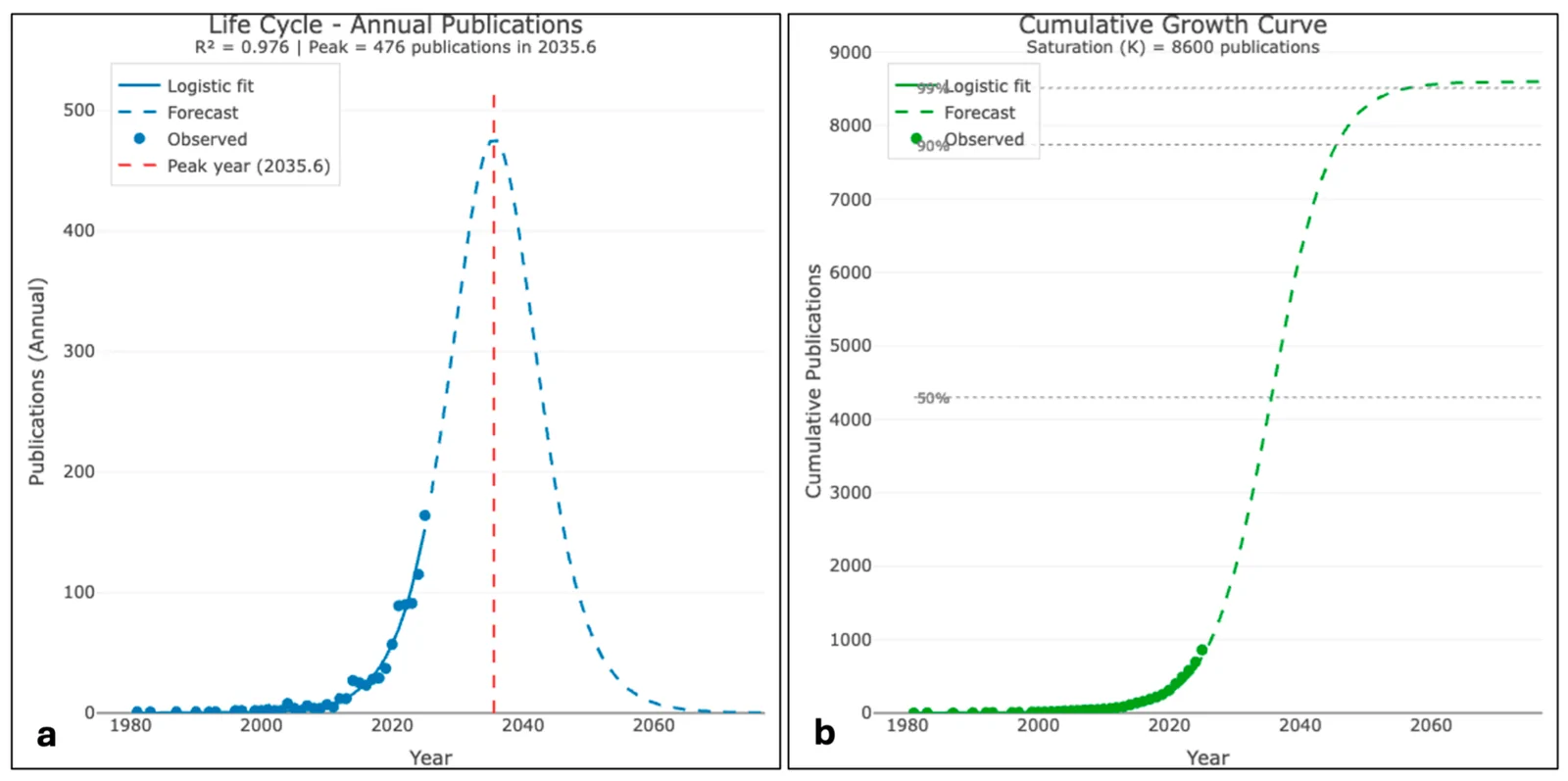
Historical and model-fitted publication growth of bacteriophage-based antimicrobial research in SEA: (**a**) annual scientific production and fitted growth trajectory and (**b**) cumulative publication growth and logistic model extension. Model-based values beyond 2025 represent extrapolations of historical trends rather than validated forecasts. Note: The labels “Forecast,” “Peak year,” and “Saturation” are terminology automatically generated by the Biblioshiny logistic growth-model output and denote mathematical characteristics of the fitted logistic function. In the present study, these labels should not be interpreted as validated predictions. “Forecast” represents extrapolation of the fitted historical trajectory beyond the observation period; “Peak year” denotes the model-derived temporal location associated with maximum modeled growth; and “Saturation” represents the estimated upper asymptote (K) of the fitted logistic function. All post-2025 estimates are conditional on the model assumptions and are presented for descriptive purposes only.

The logistic function provided a strong descriptive fit to the historical cumulative publication series (R^2^ = 0.976), reflecting the transition from a prolonged period of limited publication activity to accelerated growth, particularly after 2019 (Figure 5). The coefficient of determination describes the agreement between the fitted function and the observed historical data and should not be interpreted as evidence of out-of-sample predictive performance. Accordingly, the logistic model was used primarily to characterize the historical growth pattern rather than to forecast future publication output.

Extension of the fitted function beyond 2025 is presented only to illustrate its mathematical trajectory under the assumption that the historical growth pattern continues. The resulting asymptotic parameter and post-2025 values are model-dependent and are not interpreted as an empirically established saturation level, a predicted peak in research productivity, or a forecast of field maturity. Future publication activity may deviate substantially from the fitted trajectory because of changes in research funding, indexing coverage, scientific priorities, technological developments, regional policy, international collaboration, and publication practices. Therefore, the principal inference from the model is restricted to the observed historical pattern: bacteriophage-based antimicrobial research in SEA has undergone pronounced recent expansion, whereas the magnitude and duration of future growth remain uncertain.

### 3.2. Most Productive Authors

The distribution of the top 10 authors demonstrates that publication productivity in bacteriophage-based antimicrobial research in SEA is concentrated predominantly among researchers affiliated with Thai institutions (Table 1). These rankings are based on total publication output and therefore represent research productivity and participation within the analyzed corpus rather than citation impact or overall scientific influence. Vongkamjan of Kasetsart University leads with 21 publications, followed by Surachat of Prince of Songkla University with 20 publications. Other highly productive authors are also affiliated with Thai institutions, such as Siriraj Hospital, Chulalongkorn University, Chulabhorn Research Institute, and Walailak University. These affiliations indicate that Thailand serves as a primary regional hub for bacteriophage research. This concentration reflects a well-developed national research ecosystem with expertise spanning agricultural, environmental, clinical, and molecular microbiology applications.

Beyond Thailand, the data indicate emerging but comparatively limited contributions from Vietnam and Malaysia. Hoang of Viet Nam National University has 13 publications, while Mok of Universiti Malaysia Terengganu has 12 publications, demonstrating increasing regional participation beyond Thailand. However, the overall distribution of authors remains uneven, indicating that bacteriophage research capacity in SEA remains concentrated within a limited number of countries and institutional clusters. Enhancing cross-border collaborations, establishing shared phage biobanks, developing genomic surveillance networks, and forming translational phage-therapy consortia may broaden regional productivity and facilitate the transition from isolated institutional expertise to a more integrated Southeast Asian bacteriophage research network.

### 3.3. Most Relevant Institutions

The institutional distribution reveals a pronounced concentration of publication productivity among institutions in Thailand, Malaysia, and Singapore (Table 2). Because the ranking is based on total publication counts, these results identify the most productive participating institutions within the analyzed corpus and should not be interpreted as a ranking of institutional research impact or citation influence. Mahidol University leads with 182 publications, followed by Prince of Songkla University with 142 and Chulalongkorn University with 103, as shown in Table 2. Other Thai institutions, such as Kasetsart University, Chiang Mai University, and Walailak University, further underscore Thailand’s position as the primary regional hub for phage-related research. This concentration reflects sustained national capacity in microbiology, infectious disease research, food safety, aquaculture, and molecular biology, supported by several universities with established research pipelines.

The table also identifies significant contributions from Malaysia and Singapore, with Universiti Putra Malaysia, Universiti Sains Malaysia, the National University of Singapore, and Nanyang Technological University ranked among the top 10. These institutions demonstrate that bacteriophage research in SEA extends beyond a single national system and is concentrated within a limited number of high-output research centers. The absence of representation from many other Southeast Asian countries highlights ongoing regional disparities in research infrastructure, funding, and publication capacity. Enhancing interinstitutional networks, establishing shared phage repositories, developing genomic surveillance platforms, and fostering translational collaborations may facilitate the evolution of this institutionally concentrated field into a more integrated regional research ecosystem.

### 3.4. Most Relevant Source

The distribution of journals demonstrates that bacteriophage-based antimicrobial research in SEA is primarily published in broad-scope, internationally indexed microbiology and multidisciplinary journals. Scientific Reports leads in productivity with 30 publications, followed by Frontiers in Microbiology with 26 publications, and both PLOS One and Antibiotics with 23 publications each (Table 3). This trend highlights the interdisciplinary nature of the field, encompassing microbial genomics, antimicrobial resistance, phage biology, food safety, veterinary microbiology, and translational phage applications.

Citation metrics indicate that research visibility is influenced by factors beyond publication volume. Frontiers in Microbiology achieved the highest total citations among leading journals, whereas Veterinary World attained the highest average citations per paper. This suggests that specific veterinary or animal-health phage studies have a significant citation impact despite the lower publication output. The notable presence of MDPI journals, such as Antibiotics, Viruses, Microorganisms, and Pharmaceuticals, underscores the importance of open-access platforms in disseminating phage-related research. Collectively, these patterns position the field at the intersection of fundamental microbiology and applied antimicrobial innovation, with increasing significance for One Health, antimicrobial resistance mitigation, and regional infectious disease research.

### 3.5. Collaboration Network Analysis

**Figure 6:**
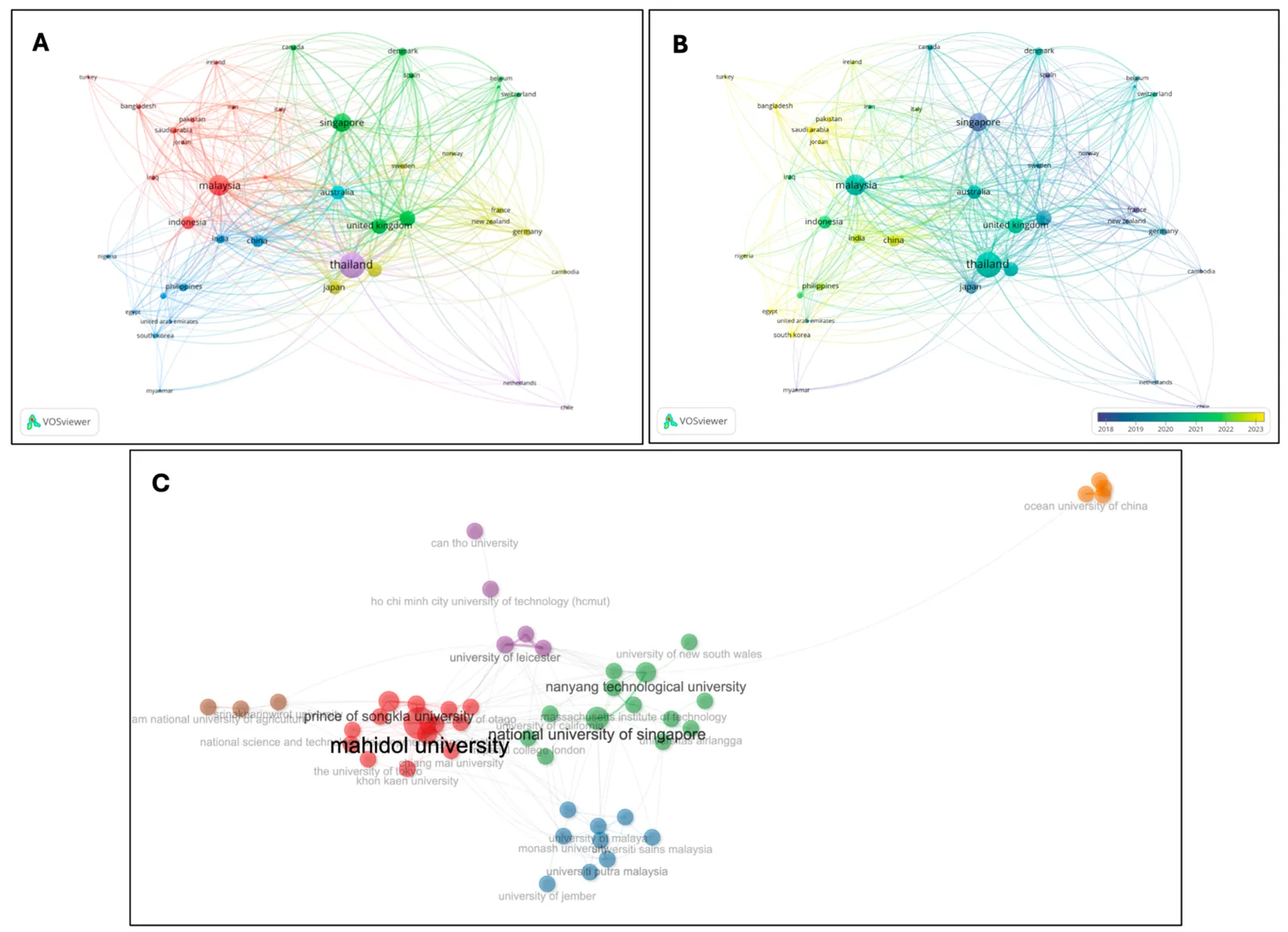
Co-authorship on bacteriophage-based antimicrobial research in SEA among countries through (**A**) network analysis, (**B**) overlay visualization, and (**C**) institutional collaboration. Node size reflects relative prominence according to the underlying VOSviewer network, colors in the network visualizations identify clusters, and links represent co-authorship relationships. In the overlay visualization, node color represents the average publication year.

The country-level co-authorship network (Figure 6A) demonstrates extensive international collaboration within the analyzed literature. Thailand, Singapore, and Malaysia are visually prominent within the network, as reflected by comparatively large nodes and numerous observable collaborative connections with regional and international partners. Thailand shows linkages with Japan, China, Australia, the United Kingdom, and other Southeast Asian countries, whereas Singapore is connected with several European and Asia-Pacific collaborators. Malaysia is similarly embedded within a broader international collaboration structure. Because formal node-level centrality measures and total link strength were not available for quantitative comparison, these patterns are interpreted descriptively and should not be considered formal rankings of network centrality.

The overlay visualization (Figure 6B) reveals the temporal development of these collaborative relationships, with node and link colors representing the average publication period from approximately 2018 to 2023. Countries shaded in blue to blue-green, including Singapore, the United Kingdom, Australia, Denmark, and several European partners, represent comparatively earlier-established contributors to the collaboration network. Thailand and Malaysia display intermediate green tones, suggesting sustained participation during the middle phase of the assessed period. More recent collaborative activity is associated with yellow-toned countries, including China, India, Pakistan, Saudi Arabia, Bangladesh, the Philippines, and several other Asian partners. This temporal distribution indicates a gradual geographical expansion of the field from established international research hubs toward broader participation by South and Southeast Asian countries, accompanied by the formation of newer regional and cross-regional research connections.

The institutional collaboration network (Figure 6C) is organized into several distinct but interconnected clusters, indicating the presence of institutionally concentrated research communities. The large red and green clusters form the principal core of the network and contain institutions with numerous intra-cluster and inter-cluster links, suggesting frequent collaboration and shared research activities among major universities and research centers. The blue and purple clusters occupy more peripheral positions but remain connected to the central network through several bridging institutions, which may facilitate knowledge exchange between otherwise specialized research groups. Smaller orange and brown clusters are located at the network margins and are connected through relatively few long-distance links, reflecting limited or project-specific participation. The uneven distribution of nodes and concentration of observable connections around a limited number of institutions indicate the presence of visually prominent collaborative hubs alongside a broader group of more peripheral or less extensively connected organizations. These patterns are interpreted descriptively and do not represent formal rankings of network centrality.

### 3.6. Keyword Co-Occurrence and Research Clusters

**Figure 7:**
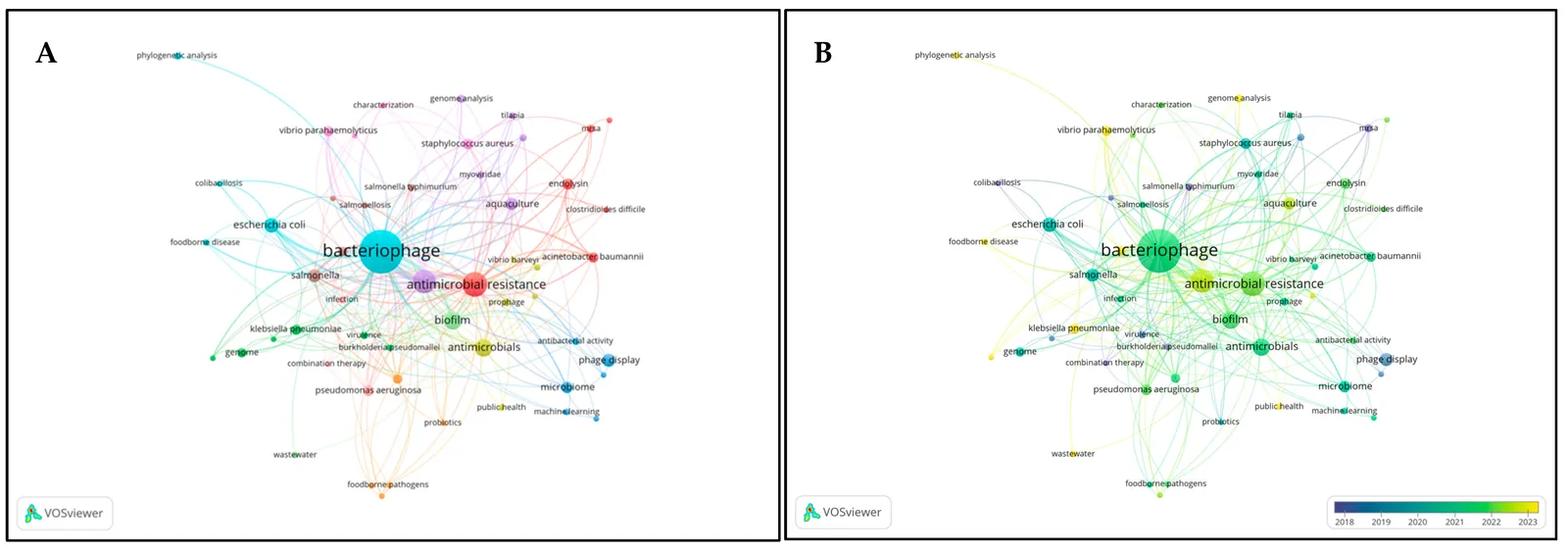
Keyword co-occurrence through (**A**) network visualization and (**B**) overlay visualization of bacteriophage-based antimicrobial research in SEA. Node size represents relative keyword occurrence, links denote keyword co-occurrence relationships, and cluster colors in (**A**) represent groups of closely associated terms. In (**B**), colors indicate the average publication year associated with each keyword. Only keywords satisfying the predefined minimum-occurrence threshold were included.

The network visualization (Figure 7A) reveals a densely interconnected intellectual structure centered on the dominant keyword “bacteriophage,” which exhibits strong co-occurrence relationships with “antimicrobial resistance,” “biofilm,” “antimicrobials,” “Escherichia coli,” “Salmonella,” and “aquaculture.” The clustering pattern indicates several overlapping research domains. One major cluster emphasizes antimicrobial resistance and therapeutic applications, linking bacteriophages with biofilms, prophages, antibacterial activity, endolysins, *A. baumannii*, and *C. difficile*. A second cluster is oriented toward foodborne and enteric pathogens, particularly *E. coli*, *Salmonella*, *Salmonella Typhimurium*, colibacillosis, salmonellosis, and foodborne disease. Additional clusters reflect phage research in aquaculture and aquatic pathogens, including *Vibrio parahaemolyticus*, *V. harveyi*, tilapia, and *S. aureus*, as well as genomic and methodological themes involving genome analysis, phylogenetic analysis, microbiomes, phage display, and machine learning. The extensive cross-cluster linkages suggest that Southeast Asian bacteriophage research integrates clinical microbiology, food safety, aquaculture, environmental surveillance, and biotechnology rather than developing as isolated thematic areas.

The overlay visualization (Figure 7B) demonstrates a temporal shift in research emphasis from foundational and methodological topics toward increasingly applied and translational themes. Earlier keywords, shown in blue to blue-green and concentrated around approximately 2018–2020, include “phage display,” “antibacterial activity,” “colibacillosis,” “*K. pneumoniae*,” “MRSA,” and aspects of *S. aureus* research, indicating an initial focus on established phage-based technologies and clinically important bacterial pathogens. Intermediate green tones characterize the central themes of bacteriophages, antimicrobial resistance, biofilms, antimicrobials, aquaculture, and several foodborne pathogens, reflecting sustained activity around 2020–2021. More recent, yellow-toned terms, particularly “genome analysis,” “phylogenetic analysis,” “*V. parahaemolyticus*,” “foodborne disease,” “public health,” “wastewater,” and “genome,” indicate growing attention to genomic characterization, molecular epidemiology, environmental reservoirs, and public-health applications. The temporal distribution also suggests that current research increasingly connects bacteriophage biology with One Health concerns, including the dissemination of antimicrobial resistance across clinical, agricultural, aquatic, and environmental systems.

### 3.7. Trend and Burning Topics

**Figure 8:**
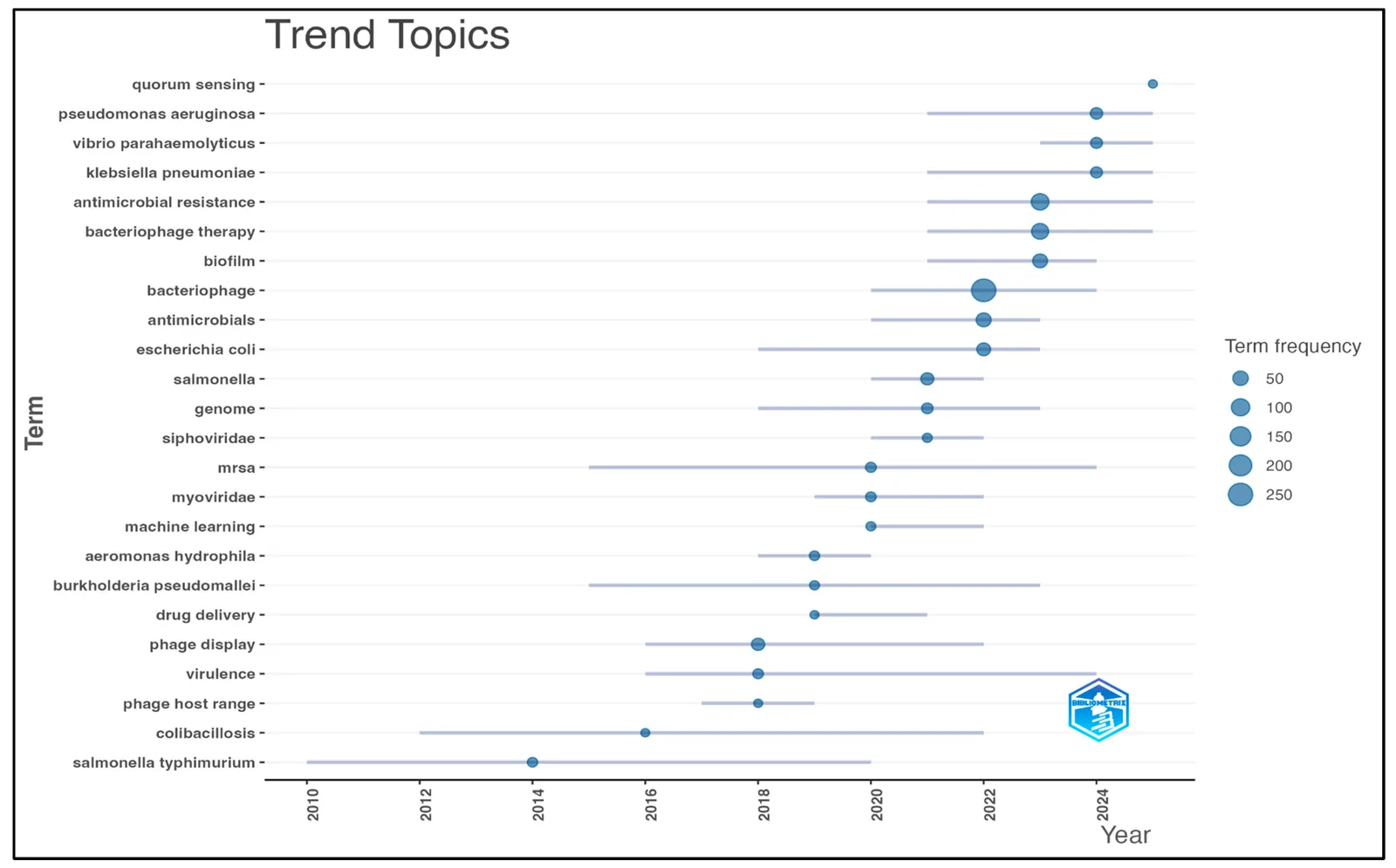
Topics studied over time in relation to bacteriophage-based antimicrobial research in SEA. Bubble size represents relative topic frequency, whereas horizontal position reflects the period during which each topic was represented in the literature. Label spacing and figure dimensions were optimized to improve the distinction among temporally overlapping topics.

The trend-topic analysis shows a progressive shift from pathogen-specific and foundational bacteriophage research toward clinically and technologically oriented themes (Figure 8). Earlier activity was concentrated on *S. Typhimurium*, colibacillosis, phage host range, virulence, and phage display, with several of these topics extending from the early or mid-2010s into the late 2010s or early 2020s. Research subsequently broadened to include *Burkholderia pseudomallei*, *Aeromonas hydrophila*, drug delivery, machine learning, and the major phage families Myoviridae and Siphoviridae. The long temporal spans of MRSA, virulence, colibacillosis, and *B. pseudomallei* indicate sustained interest, although their relatively small node sizes suggest lower overall frequencies than the dominant contemporary themes.

From approximately 2020 onward, the literature became increasingly centered on bacteriophages as antimicrobial tools against priority bacterial pathogens. “Bacteriophage” is the most frequently occurring term, with its largest bubble around 2022, while antimicrobial resistance, bacteriophage therapy, biofilm, antimicrobials, and *E. coli* emerge prominently between 2021 and 2023. More recent topics include *P. aeruginosa*, *V. parahaemolyticus*, *K. pneumoniae*, and quorum sensing, reflecting growing interest in multidrug-resistant infections, aquaculture-associated pathogens, biofilm control, and phage-bacterial communication. The contemporaneous prominence of genome-related research further indicates an increasing reliance on genomic characterization to assess phage identity, host specificity, safety, and therapeutic potential.

**Figure 9:**
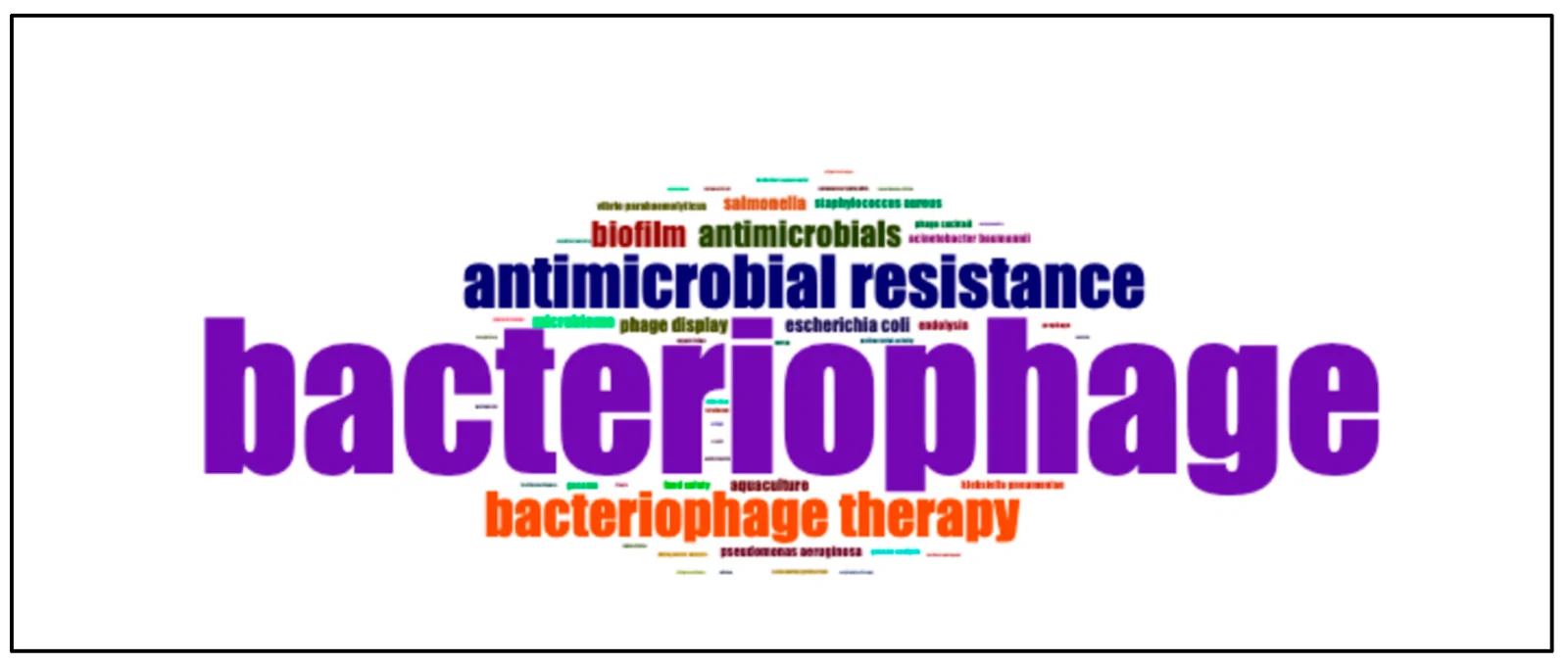
Word cloud visualizes the most frequently occurring terms in bacteriophage-based antimicrobial research in SEA, with themes in the field. Font size represents the relative frequency of terms within the analyzed corpus. The visualization emphasizes the most recurrent concepts to facilitate graphical interpretation; quantitative thematic relationships are evaluated separately through the keyword co-occurrence and conceptual-structure analyses.

The word cloud identifies “bacteriophage” as the most frequently occurring term and the principal conceptual focus of Southeast Asian bacteriophage research (Figure 9). Its pronounced visual dominance, together with the prominence of “antimicrobial resistance” and “bacteriophage therapy,” indicates that the literature is strongly oriented toward the therapeutic use of phages as alternatives or adjuncts to conventional antibiotics. Other visible terms, including “biofilm,” “antimicrobials,” “antibacterial activity,” and “drug resistance,” further reflect sustained interest in controlling difficult-to-treat bacterial infections, particularly those involving multidrug-resistant or biofilm-forming pathogens. The relative prominence of these terms suggests that clinical and translational applications constitute a major thematic core of the regional research landscape.

A second thematic layer is represented by pathogen-, environment-, and application-specific terms such as “*Salmonella*,” “*E. coli*,” “*S. aureus*,” “*P. aeruginosa*,” “*K. pneumoniae*,” “aquaculture,” and “phage display.” These terms indicate that bacteriophage investigations in SEA span food safety, human and animal health, aquatic production systems, and biotechnology. The presence of organism-specific keywords reflects considerable attention to bacterial pathogens of medical, veterinary, and economic importance, whereas terms related to genome analysis, characterization, and host interactions point to the continued role of molecular and genomic approaches in phage identification and functional assessment. The overall term distribution portrays a field structured around antimicrobial resistance mitigation, phage-based treatment, pathogen control, and the characterization of bacteriophages for clinical, agricultural, and environmental applications.

### 3.8. Conceptual Structure Mapping

**Figure 10:**
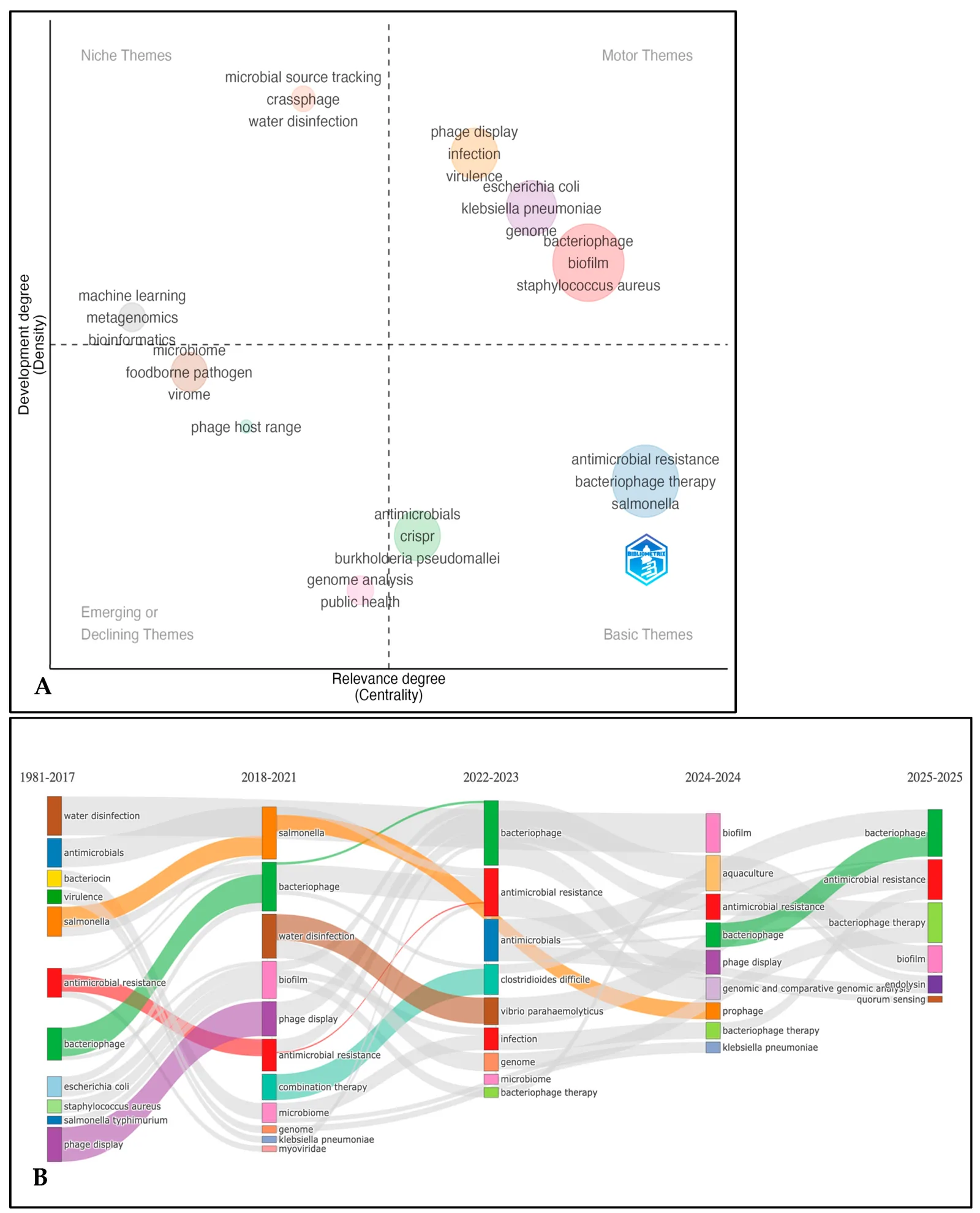
Strategic thematic map (**A**) and thematic evolution (**B**) of bacteriophage-based antimicrobial research in SEA.

The strategic thematic map (Figure 10A) identifies a differentiated knowledge structure based on thematic centrality and density. The motor-theme quadrant contains a highly developed and influential cluster centered on bacteriophage, biofilm, *S. aureus*, genome, *K. pneumoniae*, *E. coli*, virulence, infection, and phage display, indicating that phage-host interactions, pathogen control, genomic characterization, and biofilm-associated infections constitute mature and structurally important research fronts. By contrast, antimicrobial resistance, bacteriophage therapy, and Salmonella occupy the basic-theme quadrant, reflecting high relevance across the field but comparatively lower internal development. Their large cluster size indicates substantial publication activity and broad integration with other topics, particularly the therapeutic use of phages against resistant and foodborne pathogens.

The remaining quadrants reveal specialized and transitional areas of inquiry. The niche-theme cluster comprising microbial source tracking, crAssphage, and water disinfection is highly developed but weakly connected to the broader research structure, suggesting methodological specialization in environmental monitoring and water-quality assessment. A second upper-left grouping involving machine learning, metagenomics, bioinformatics, and microbiome lies near the centrality boundary, indicating an increasingly developed computational and omics-oriented domain that has not yet become fully integrated with the field’s main therapeutic agenda. Emerging or declining themes include foodborne pathogen, virome, phage host range, antimicrobials, CRISPR, *B. pseudomallei*, genome analysis, and public health; their lower density and centrality suggest either recent development, limited consolidation, or declining relative emphasis within the Southeast Asian research landscape.

The thematic evolution analysis (Figure 10B) shows substantial reorganization across five periods. During 1981–2017, the field was distributed among water disinfection, antimicrobials, bacteriocins, virulence, *Salmonella*, antimicrobial resistance, bacteriophages, *E. coli*, *S. aureus*, *S. Typhimurium*, and phage display. Between 2018 and 2021, these topics converged into broader themes involving bacteriophages, biofilms, antimicrobial resistance, combination therapy, microbiomes, genomes, *K. pneumoniae*, and Myoviridae. In 2022–2023, bacteriophage and antimicrobial resistance became dominant, accompanied by antimicrobials, *C. difficile*, *Vibrio parahaemolyticus*, infection, genome, microbiome, and bacteriophage therapy. The 2024 period further emphasized biofilm, aquaculture, antimicrobial resistance, bacteriophage, phage display, comparative genomics, prophage, bacteriophage therapy, and *K. pneumoniae*, while the 2025 themes consolidated around bacteriophage, antimicrobial resistance, bacteriophage therapy, biofilm, endolysin, genomic and comparative genomic analysis, and quorum sensing.

**Figure 11:**
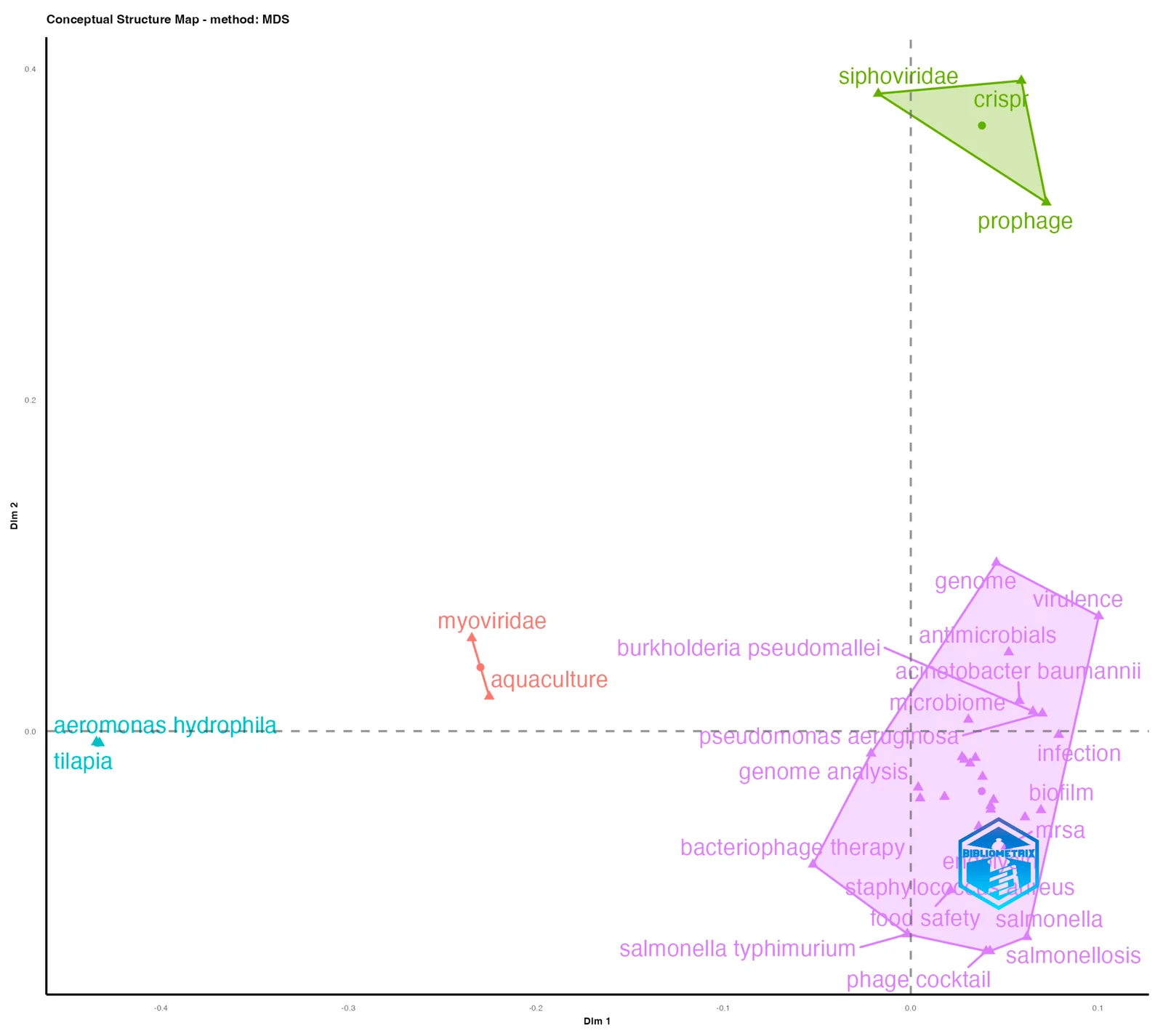
Factorial analysis through four-clustered multidimensional scaling (MDS) of bacteriophage-based antimicrobial research in SEA. Spatial proximity represents conceptual similarity among terms, whereas greater separation indicates comparatively distinct thematic orientations. Colors distinguish the four identified clusters. Figure dimensions and label placement were optimized to reduce overlap while preserving cluster membership and relative spatial relationships. Because Dimensions 1 and 2 represent latent dimensions derived from similarity relationships rather than predefined biological variables, interpretation emphasizes relative proximity, separation, and cluster composition.

The four-cluster multidimensional scaling (MDS) map reveals a clearly differentiated yet partially interconnected conceptual structure of bacteriophage-based antimicrobial research in SEA (Figure 11). In this representation, terms positioned closer to one another share stronger conceptual similarity or co-occurrence patterns, whereas greater spatial separation indicates more distinct research orientations.

The largest and most internally diverse cluster, shown in purple and concentrated around the lower-right portion of the map, represents the principal translational and clinical antimicrobial research domain. It includes terms such as bacteriophage therapy, phage cocktail, biofilm, infection, MRSA, *S. aureus*, *Salmonella*, salmonellosis, *P. aeruginosa*, *A. baumannii*, *B. pseudomallei*, antimicrobials, virulence, microbiome, genome, and genome analysis. The close proximity of these terms indicates that the regional literature is strongly organized around the application of bacteriophages against clinically and epidemiologically important bacterial pathogens, particularly those associated with antimicrobial resistance, biofilm formation, and difficult-to-treat infections. The co-location of genomic and microbiome-related terms with therapeutic concepts further suggests increasing integration of molecular characterization, pathogen genomics, and microbial ecology into phage-based antimicrobial development. Within this cluster, the association of phage cocktail with food safety, *Salmonella*, and *salmonellosis* also demonstrates that translational phage research extends beyond human clinical infections to foodborne-pathogen control and food-safety applications.

A second, comparatively compact cluster located in the upper-right quadrant is characterized by *Siphoviridae*, CRISPR, and prophage. Its marked separation from the major therapeutic cluster indicates a conceptually specialized line of investigation focused on phage biology, lysogeny, bacterial-phage evolutionary interactions, and molecular defense systems rather than direct antimicrobial application. The association between prophage and CRISPR is particularly meaningful because these concepts concern the genomic consequences of phage integration and bacterial adaptive immunity, while the presence of *Siphoviridae* reflects taxonomic and structural characterization of temperate or siphovirus-like bacteriophages. This cluster therefore represents a more fundamental molecular and genomic research axis that complements, but remains distinguishable from, therapeutic phage research. A third cluster, positioned toward the center-left and represented by *Myoviridae* and aquaculture, reflects an applied aquatic-health research theme. Their close positioning suggests that myovirus-type bacteriophages have featured prominently in studies addressing bacterial disease control in aquaculture systems. This finding is consistent with the regional importance of aquaculture and indicates that bacteriophage research in SEA has developed not only around medical applications but also around disease management in economically important aquatic production systems.

The fourth cluster, situated farthest to the left, contains *A. hydrophila* and *tilapia* and is the most spatially isolated conceptual domain in the map. This strong separation suggests a highly specialized aquaculture-oriented research niche centered on phage-mediated control or characterization of *A. hydrophila*, an important bacterial pathogen of freshwater fish, particularly tilapia. Although this cluster is conceptually related to the adjacent aquaculture*-Myoviridae* theme, its greater distance from the map’s central therapeutic domain indicates limited integration with the broader literature on human pathogens, antimicrobial resistance, and clinical phage therapy. Taken together, the MDS configuration demonstrates that bacteriophage-based antimicrobial research in SEA is organized around four principal knowledge domains: a dominant translational antimicrobial and pathogen-control axis; a molecular phage-host interaction and CRISPR/prophage axis; a broader aquaculture-phage application axis; and a highly specialized *A. hydrophila-tilapia* axis. The relative concentration of terms in the purple cluster suggests that clinically oriented and One Health-relevant applications constitute the conceptual core of the field, while the more peripheral clusters represent specialized but potentially complementary research trajectories. Importantly, the MDS dimensions themselves should be interpreted as latent axes derived from similarity relationships rather than as predefined biological variables; thus, the strongest inference arises from relative distances, clustering, and thematic composition rather than from assigning fixed meanings to Dimensions 1 and 2.

## 4. Discussion

The bibliometric patterns identified in this study demonstrate substantial growth in the scientific productivity and scholarly visibility of bacteriophage-based antimicrobial research in SEA, as reflected by increasing publication output, citation activity, international collaboration, and thematic diversification. The marked acceleration in publications after 2019 parallels the broader global resurgence of interest in bacteriophages as potential precision antimicrobial agents for multidrug-resistant infections, biofilm-associated disease, and other therapeutic challenges associated with declining antibiotic effectiveness [11,16]. Importantly, however, increasing publication volume should not be interpreted as direct evidence of corresponding clinical advancement or translational readiness. Bibliometric indicators quantify research activity, scholarly influence, and the organization of scientific knowledge but do not measure therapeutic efficacy, progression through clinical trial phases, regulatory approval, manufacturing readiness, or implementation in routine healthcare. Thus, the observed growth primarily demonstrates increasing regional scientific engagement with bacteriophage-based antimicrobial approaches. Whether this expanding research base translates into clinically deployable phage therapies depends on separate translational milestones, including robust preclinical validation, controlled clinical evaluation, standardized manufacturing, regulatory frameworks, and healthcare implementation [17].

The concentration of highly productive authors and institutions in Thailand, alongside significant contributions from Singapore and Malaysia, indicates the emergence of regional centers of excellence advancing bacteriophage innovation in SEA. These centers benefit from established microbiology infrastructure, robust infectious disease programs, and sustained investment in molecular biology, food safety, and aquaculture research. Concurrently, the collaboration network reveals that Southeast Asian bacteriophage research is increasingly integrated into international scientific partnerships with the United States, China, Japan, Australia, and several European countries. This global integration is essential, as effective clinical translation of phage therapy requires access to comprehensive phage collections, genomic characterization platforms, standardized manufacturing processes, and multicenter clinical validation studies [10]. Recent analyses underscore that the future of therapeutic phage development will rely heavily on collaborative networks that support personalized phage selection [18]. Therefore, strengthening intra-ASEAN partnerships, regional phage repositories, and coordinated surveillance programs may facilitate the transition from institutionally concentrated expertise to a more integrated Southeast Asian phage research ecosystem.

The keyword co-occurrence network, trend-topic analysis, and thematic mapping collectively indicate that bacteriophage research in SEA is increasingly focused on antimicrobial resistance, biofilm control, and clinically significant bacterial pathogens. The strong associations among *P. aeruginosa*, *K. pneumoniae*, *A. baumannii*, *S. aureus*, biofilm, and antibiotic resistance demonstrate that phages are being investigated as targeted interventions against some of the most challenging multidrug-resistant organisms. These findings align with global research priorities, which emphasize phage–antibiotic synergy, antibiofilm therapies, and pathogen-specific treatment strategies. Experimental and clinical evidence has established that phages can disrupt mature biofilms, enhance antibiotic susceptibility, and reduce bacterial virulence, thereby improving outcomes against otherwise persistent infections [19,20].

A key aspect of the thematic evolution identified in this study is the growing integration of environmental and One Health perspectives into bacteriophage research. Emerging themes such as wastewater surveillance, metagenomics, microbial source tracking, food safety, aquaculture, and environmental microbiology indicate that phage investigations are extending beyond traditional clinical applications into interconnected ecological systems. In SEA, where intensive aquaculture, high urban density, and widespread antimicrobial use create complex pathways for AMR transmission, these interdisciplinary approaches are especially pertinent. Recent One Health studies have identified wastewater systems as critical reservoirs and surveillance points for antimicrobial resistance genes, resistant bacteria, and bacteriophages that influence microbial ecology and the dissemination of resistance [21,22]. Advances in metagenomics and environmental viromics have enabled the discovery of novel phages from previously unexplored ecosystems and enhanced understanding of phage-host dynamics within complex microbial communities [23].

The factorial analysis identifies several emerging research fronts likely to shape the future direction of bacteriophage research in SEA. While clinical AMR- and biofilm-focused studies remain predominant, the emergence of CRISPR-associated technologies, genome analysis, endolysins, and aquaculture-centered phage applications reflects a broadening of research priorities. The presence of CRISPR in a distinct thematic cluster is particularly noteworthy, as phage-assisted CRISPR systems are being explored as precision antimicrobial tools that can selectively eliminate resistant bacteria or remove resistance determinants with minimal impact on beneficial microbiota [24,25]. Similarly, the rise of endolysin-related research indicates increasing interest in phage-derived enzymes as alternative antibacterial agents with strong activity against multidrug-resistant pathogens [26]. Aquaculture-focused themes involving *A. hydrophila* and tilapia highlight the growing significance of phage biocontrol strategies for sustainable food production and disease management in key aquatic systems.

### 4.1. Infrastructure Deficiency in Phage Biobanking and Genomic Surveillance

The thematic analyses demonstrate increasing attention to phage therapy, genome analysis, biofilm control, antimicrobial resistance, and clinically important bacterial pathogens, indicating that the regional literature is becoming increasingly application-oriented. However, these bibliometric patterns measure research attention rather than translational readiness. The dataset does not directly evaluate the availability of phage biobanks, GMP-compliant manufacturing, regulatory approval pathways, clinical trial infrastructure, or therapeutic implementation. Accordingly, the prominence of clinically relevant themes should be interpreted as evidence of increasing scientific engagement with translational questions rather than evidence that corresponding clinical capacity has already been established.

Published literature outside the bibliometric indicators identifies standardized phage characterization, genomic quality control, manufacturing capacity, regulatory frameworks, and clinical validation as important requirements for therapeutic translation [27,28,29,30,31,32,33,34,35,36,37,38]. In the context of the present results, these considerations provide a framework for interpreting the gap between increasing scientific productivity and clinical implementation but should not be considered outcomes demonstrated by the bibliometric analysis itself. Future assessments integrating bibliometric data with clinical trials, regulatory approvals, patents, manufacturing infrastructure, and therapeutic-use indicators would enable a more direct evaluation of SEA’s translational phage capacity.

Fragmentation of existing phage collections and the lack of centralized phage biobanking systems significantly contribute to the translational gap. Despite over a century of global phage discovery, substantial fragmentation persists in the cataloging, preservation, and coordination of phage resources, leading to inefficiencies in accessing and utilizing available collections [27,29]. Phage biobanks serve as structured repositories that store, catalog, characterize, and distribute well-defined bacteriophages for research, clinical, industrial, and public health purposes. These repositories are increasingly envisioned as dynamic platforms that integrate genomic, phenotypic, ecological, and host-range data to enable rapid phage selection and precision therapeutic applications [30,31,32]. Countries such as Australia and Israel have addressed these challenges through national initiatives, such as the Israeli Phage Bank (IPB), which houses more than 300 bacteriophages targeting 16 bacterial pathogens and provides a centralized framework for phage characterization and clinical use [30,32]. However, no comparable ASEAN-wide phage repository currently exists, despite the region’s rich microbial diversity and increasing research activity.

The absence of coordinated genomic surveillance further exacerbates these challenges. Contemporary phage development depends on whole-genome sequencing, comparative genomics, metagenomics, and advanced bioinformatics to characterize phage diversity, predict host specificity, identify undesirable genetic elements, and monitor phage-host evolutionary dynamics. However, sequencing infrastructure and bioinformatics capacity are unevenly distributed across SEA, which may contribute to the institutional and geographic disparities identified in this study. As regional research increasingly focuses on antimicrobial resistance surveillance, genome-informed phage discovery, microbiome research, and precision phage applications, the creation of integrated phage biobanks and genomic surveillance networks will become essential. Therefore, establishing ASEAN-wide phage repositories, shared genomic databases, and collaborative surveillance infrastructures should be prioritized to accelerate the clinical translation of bacteriophage-based interventions and enhance regional preparedness against antimicrobial resistance [27,28,29].

### 4.2. Translational Readiness and Innovation Capacity of Southeast Asian Phage Research

The expansion of bacteriophage-based antimicrobial publications in SEA demonstrates growing scientific capacity and engagement, but this should be distinguished from translational readiness. Scientific productivity reflects the generation and dissemination of research, whereas translational readiness requires progression through additional stages, including reproducible preclinical efficacy, toxicological and pharmacological evaluation, clinical trials, standardized phage characterization, scalable manufacturing, regulatory approval, and integration into healthcare systems. The present bibliometric indicators directly evaluate the former but cannot independently establish the latter. Consequently, the increasing prominence of phage therapy, genomic surveillance, One Health frameworks, and biotechnology-oriented themes should be interpreted as evidence of growing research attention and potential translational direction, rather than as confirmation that these technologies have achieved clinical maturity.

Available evidence nevertheless indicates that SEA is developing several scientific components that could support future translation, including increasing expertise in genomic surveillance, molecular epidemiology, AMR research, and One Health approaches [33,34]. Emerging attention to bacteriophage therapy, resistome surveillance, artificial intelligence-assisted antimicrobial discovery, and precision genomic monitoring further demonstrates diversification toward research areas with translational potential [33,35]. However, substantial structural barriers, including unequal institutional capacity, fragmented funding, limited data sharing, inconsistent regulatory frameworks, weak academia–industry integration, and underdeveloped environmental surveillance, continue to constrain the progression of research outputs into deployable interventions [21,36,37]. Therefore, the bibliometric expansion documented in this study is best interpreted as growth in the regional knowledge base and scientific research capacity, while actual therapeutic development should be evaluated independently through indicators such as clinical trial activity, GMP manufacturing capacity, regulatory pathways, technology transfer, commercialization, and documented clinical implementation.

Despite recent advancements, significant structural and translational barriers continue to limit the region’s readiness to develop a mature antimicrobial innovation ecosystem. Collaborative AMR research in Asia is constrained by unequal institutional capacities, fragmented funding structures, limited data-sharing mechanisms, inconsistent regulatory frameworks, and weak academia-industry partnerships, which often prevent promising discoveries from advancing beyond proof-of-concept stages [36]. Studies of AMR collaborations across Asia have identified persistent inequities in research leadership, access to infrastructure, and the sustainability of long-term partnerships, collectively impeding the development of coordinated innovation pipelines [36,37]. Furthermore, environmental AMR surveillance and resistome monitoring remain underdeveloped in many Southeast Asian countries, despite growing recognition that environmental reservoirs are central drivers of the emergence and dissemination of resistance [21]. Recent regional assessments highlight substantial gaps in cross-sector integration, implementation science, technology transfer, and commercialization capacity, demonstrating that scientific productivity alone is insufficient to establish sustainable antimicrobial innovation ecosystems [37]. Therefore, future progress will require stronger transnational research networks, harmonized regulatory and governance frameworks, increased biotechnology investment, and integrated platforms that connect surveillance systems, academic institutions, healthcare sectors, environmental monitoring programs, and industrial stakeholders into coordinated translational pathways to accelerate antimicrobial discovery, implementation, and regional health security [33,37].

### 4.3. One Health Integration and Ecological AMR Containment

The thematic analyses demonstrate that bacteriophage-based antimicrobial research in SEA extends beyond clinical applications to food safety, aquaculture, wastewater, microbial source tracking, environmental microbiology, and public health. The increasing prevalence of these themes supports a growing One Health orientation in which phage research intersects with human, animal, food production, and environmental systems. Aquaculture-related pathogens and environmental surveillance themes were particularly evident across the keyword, thematic-evolution, and conceptual-structure analyses. These patterns are relevant to SEA because antimicrobial resistance occurs across interconnected ecological compartments [4,21,22].

Nevertheless, the bibliometric evidence identifies the prominence and relationships of these research topics rather than demonstrating the effectiveness of phage-based ecological interventions. The results therefore indicate thematic diversification toward One Health applications, while questions concerning efficacy, ecological safety, and implementation require experimental and translational investigation.

Phage-based interventions are increasingly recognized as ecological containment systems that interrupt the emergence, persistence, and dissemination of AMR across interconnected human, agricultural, and environmental interfaces within a One Health framework. In contrast to broad-spectrum antibiotics, bacteriophages demonstrate highly specific bactericidal activity, selectively targeting resistant bacterial populations while minimizing disruption of beneficial microbiota and ecosystem microbial functions [8,19]. This ecological precision allows phages to reduce reservoirs of multidrug-resistant pathogens in hospitals, livestock production systems, aquaculture operations, and food-processing environments without exerting the broad selective pressures commonly associated with antibiotic use [20,38]. Additionally, phages can penetrate and disrupt bacterial biofilms, which act as critical ecological reservoirs of resistant microorganisms and facilitate horizontal gene transfer among bacterial populations [39,40]. By reducing biofilm biomass and suppressing resistant bacterial communities, phage-based interventions may decrease the persistence and transmission of antimicrobial resistance genes (ARGs) across environmental compartments, thereby limiting the movement of resistance determinants between humans, animals, and surrounding ecosystems [20,40].

In addition to direct bacterial elimination, phages may serve as dynamic ecological regulators that reshape microbial community structures and weaken environmental feedback loops sustaining AMR dissemination. Wastewater treatment plants, agricultural runoff systems, manure-amended soils, and aquatic environments are increasingly identified as critical interfaces where resistant bacteria and ARGs circulate among human, animal, and environmental reservoirs [4,41]. Targeted phage application in these settings has been proposed as a strategy to reduce antibiotic-resistant bacterial loads before reintroduction into clinical and agricultural systems via contaminated water, food products, or environmental exposure pathways [19,20]. However, ecological deployment requires careful genomic characterization of candidate phages to exclude lysogenic elements and ARG-associated sequences, as temperate phages can occasionally facilitate transduction-mediated gene transfer under specific conditions [42,43]. Therefore, contemporary phage-based containment approaches emphasize the use of strictly lytic phages, rationally designed phage cocktails, and surveillance-guided implementation strategies to maximize bacterial control while minimizing evolutionary risks [8,38]. When integrated with antimicrobial stewardship, environmental surveillance, and One Health biosecurity frameworks, phage technologies represent a promising ecological tool for suppressing AMR transmission networks and enhancing resilience against the global spread of antimicrobial resistance [4,38].

## 5. Future Directions and Research Priorities

ASEAN countries could enhance regional collaboration in phage research by integrating phage innovation into the broader regional response to antimicrobial resistance (AMR) and the One Health framework, connecting human, animal, food-production, and environmental reservoirs of AMR in SEA and underscoring the importance of coordinated research and surveillance over isolated national initiatives [44,45]. Regional networks can facilitate the sharing of phage resources, bacterial isolates, genomic data, technical expertise, and research infrastructure. Such collaboration is particularly valuable because translating phage research requires standardized characterization, safety assessment, and clinical evaluation beyond initial phage discovery [11,46]. Future regional research should also consider developing shared phage biobanks, genomic surveillance resources, and multi-country research platforms. These resources would support studies targeting priority multidrug-resistant pathogens, including carbapenem-resistant Enterobacterales, *P. aeruginosa*, *A. baumannii*, and *S. aureus*, while enabling more systematic evaluation of phage–host interactions and therapeutic applications [47,48,49,50]. However, the bibliometric findings of this study reflect research trends and emerging scientific priorities, rather than the clinical or regulatory readiness of individual ASEAN countries. Future investigations should therefore supplement bibliometric indicators with measures of translational activity, such as clinical trials, patents, regulatory approvals, manufacturing capacity, technology transfer, and documented therapeutic applications. Such analyses would clarify whether corresponding advances toward practical application accompany the observed increase in scientific productivity and thematic diversification. Regulatory heterogeneity, manufacturing requirements, and limited clinical evidence remain important considerations for future translational assessment and require dedicated evaluation beyond bibliometric analysis [11,46,51].

## 6. Limitations of the Study

Several limitations must be acknowledged when interpreting these findings. First, the analysis relied exclusively on Scopus-indexed publications. While Scopus offers broad coverage and standardized bibliographic metadata suitable for bibliometric analysis, relevant studies published in local, regional, non-indexed, or non-English-language journals may have been excluded. This limitation is significant in SEA, where research from institutions with lower international visibility is often disseminated through national or institutional journals not indexed by Scopus. Therefore, differences in publication productivity among countries should be interpreted as patterns of Scopus-indexed scientific output and visibility, not as comprehensive measures of total regional research activity.

Second, the dataset’s composition and thematic structure depend on the search strategy. The query required that bacteriophage-related terminology appear in the context of antimicrobial, antibacterial, infection, AMR, MDR, or biofilm, because the objective was to characterize bacteriophage-based antimicrobial research rather than all aspects of phage biology. This specificity may have excluded fundamental studies of phage ecology, taxonomy, evolution, environmental virology, phage–host interactions, or genomics that did not explicitly use antimicrobial-context terminology. Consequently, the thematic prominence of AMR, phage therapy, biofilms, pathogen control, aquaculture, and related topics partly reflects the defined analytical scope. It must not be extrapolated to the complete bacteriophage literature of SEA. The study therefore makes inferences only about the antimicrobial-oriented phage research corpus captured by the query. A broader bacteriophage-focused bibliometric analysis, ideally with formal sensitivity analyses of publication volume, country and institutional rankings, and thematic structure under alternative search formulations, would be valuable for determining how the intellectual structure of the complete regional phage literature differs from the antimicrobial-focused landscape characterized here.

Third, bibliometric growth indicators and model-based extrapolations should not be interpreted as measures or predictions of clinical progress. The logistic growth model was derived from historical publication counts and does not account for external factors that may affect future scientific production. Consequently, extrapolated publication levels and timing represent mathematical extensions of the observed trajectory, not validated forecasts. Furthermore, publication counts, citation indicators, collaboration networks, and thematic prominence measure scientific activity and scholarly influence but do not directly assess therapeutic efficacy, clinical trial progression, regulatory approval, manufacturing readiness, commercialization, or implementation in healthcare practice. The increasing volume of phage-related publications and the growing prominence of translational themes indicate expanding scientific attention and research capacity rather than demonstrated clinical advancement. Future assessments could complement bibliometric indicators with translational metrics, including registered clinical trials, therapeutic-use cases, patents, regulatory approvals, GMP-compliant manufacturing capacity, and documented clinical outcomes.

An additional limitation concerns the quantitative characterization of bibliometric networks. Although VOSviewer enabled visualization of co-authorship, institutional collaboration, and keyword co-occurrence structures, node-level parameters such as total link strength and formal centrality measures were not consistently available from the final analytical outputs and could not be reliably reconstructed retrospectively. Network interpretations were therefore primarily descriptive and based on observable patterns of node prominence, connectivity, clustering, and inter-node relationships. Consequently, terms implying formal graph-theoretic centrality were avoided unless supported by quantitative measures. Future studies that retain complete network output files could complement visual science mapping by including total link strength, degree, betweenness, closeness, or other network metrics to provide a more quantitative assessment of collaborative and conceptual structures.

## 7. Conclusions

Over the past decade, bacteriophage-based antimicrobial research in SEA has expanded significantly, evolving into a multidisciplinary field that encompasses clinical microbiology, food safety, agriculture, aquaculture, environmental surveillance, and One Health. The antimicrobial-focused literature analyzed in this study has shifted from primarily pathogen-specific, laboratory-based investigations to include phage therapy, biofilm control, genomic characterization, foodborne pathogen control, aquatic animal health, wastewater and environmental applications, and increasingly integrated One Health approaches. These trends indicate that regional bacteriophage research contributes not only as a potential therapeutic alternative to antibiotics but also as a tool for pathogen biocontrol, sustainable food and aquaculture production, environmental monitoring, microbial source tracking, and the management of antimicrobial-resistant bacteria across interconnected human, animal, food-production, and environmental systems. However, these findings should be interpreted within the specific scope of the search strategy, which focused on bacteriophage research in antimicrobial or bacterial-control contexts and does not capture the full spectrum of fundamental phage biology in SEA. Likewise, the observed increase in publication activity and the projected extension of historical growth patterns should not be regarded as evidence of future publication rates or equivalent progress in clinical translation. Advancing regional development will require enhanced cross-sector and cross-country collaboration, shared phage and genomic resources, standardized research frameworks, and greater integration of clinical, veterinary, agricultural, aquaculture, food-safety, and environmental surveillance systems. From a broader One Health perspective, bacteriophage research constitutes not only an emerging antimicrobial therapeutic platform but also a potentially valuable element of integrated strategies for bacterial disease control, sustainable production, environmental health, and antimicrobial resistance mitigation across SEA.

## Figures and Tables

**Table 1:** Top 10 authors publishing research on bacteriophage-based antimicrobial research in SEA.

Author Name	Affiliation	Country	TP
Vongkamjan, Kitiya	Kasetsart University	Thailand	21
Surachat, Komwit	Prince of Songkla University	Thailand	20
Chaicumpa, Wanpen	Siriraj Hospital	Thailand	14
Chaikeeratisak, Vorrapon	Chulalongkorn University	Thailand	14
Hoang, Hoang Anh	Viet Nam National University	Vietnam	13
Korbsrisate, Sunee	Siriraj Hospital	Thailand	13
Mok, Wenjye	Universiti Malaysia Terengganu	Malaysia	12
Mongkolsuk, Skorn	Chulabhorn Research Institute	Thailand	12
Pelyuntha, Wattana	Walailak University	Thailand	12
Sirikanchana, Kwanrawee	Chulabhorn Research Institute	Thailand	12

Abbreviation: TP: Total publications.

**Table 2:** Top 10 institutions publishing research on bacteriophage-based antimicrobial research in SEA.

Rank	Affiliation	Country	TP
1st	Mahidol University	Thailand	182
2nd	Prince of Songkla University	Thailand	142
3rd	Universiti Putra Malaysia	Malaysia	139
4th	National University of Singapore	Singapore	112
5th	Chulalongkorn University	Thailand	103
6th	Nanyang Technological University	Singapore	73
7th	Kasetsart University	Thailand	64
8th	Chiang Mai University	Thailand	50
9th	Walailak University	Thailand	37
10th	Universiti Sains Malaysia	Malaysia	33

Abbreviation: TP: Total publications.

**Table 3:** Top 10 Scopus-indexed journals publishing research on bacteriophage-based antimicrobial research in SEA.

Rank	Journal Title	CiteScore 2024	Publisher	TP	TC	ACP	h-Index
1st	Scientific Reports	6.7	Springer Nature	30	740	24.67	17
2nd	Frontiers in Microbiology	8.5	Frontiers Media S.A.	26	963	37.04	16
3rd	PLOS One	5.4	Public Library of Science	23	478	20.78	12
4th	Antibiotics	8.7	Multidisciplinary Digital Publishing Institute (MDPI)	23	817	35.52	13
5th	Viruses	7.7	Multidisciplinary Digital Publishing Institute (MDPI)	17	359	21.12	11
6th	Phage Therapy Applications and Research	5.3	SAGE	11	102	9.27	6
7th	Microbiology Spectrum	5.8	American Society for Microbiology	12	128	10.67	7
8th	Microorganisms	7.7	Multidisciplinary Digital Publishing Institute (MDPI)	10	176	17.60	8
9th	Veterinary World	4.2	Veterinary World	9	570	63.33	6
10th	Pharmaceuticals	7.7	Multidisciplinary Digital Publishing Institute (MDPI)	9	231	25.67	8

Abbreviation: TP: Total publications; TC: Total citations; ACP: Average citations per paper.

## Data Availability

The data analyzed in this study were obtained from Scopus under an institutional license. Restrictions apply to the availability of these raw data, which are not publicly accessible due to vendor policy. The exact search queries and processed data supporting the findings are available from the corresponding author upon reasonable request.
